# Ready for translation: non-invasive auricular vagus nerve stimulation inhibits psychophysiological indices of stimulus-specific fear and facilitates responding to repeated exposure in phobic individuals

**DOI:** 10.1038/s41398-025-03352-0

**Published:** 2025-04-09

**Authors:** Christoph Szeska, Kai Klepzig, Alfons O. Hamm, Mathias Weymar

**Affiliations:** 1 University of Potsdam, Department of Biological Psychology and Affective Science, Karl-Liebknecht-Str. 24-25, Potsdam, Germany; 2 University of Greifswald, Department of Physiological and Clinical Psychology / Psychotherapy, Franz-Mehring-Strasse 47, Greifswald, Germany; 3 University Medicine Greifswald, Center for Diagnostic Radiology and Neuroradiology, Functional Imaging Unit, Greifswald, Germany

**Keywords:** Long-term memory, Human behaviour, Psychiatric disorders

## Abstract

Recent laboratory research showed that vagus nerve stimulation promotes fear extinction, the inhibitory core mechanism of exposure treatment, presumably via activation of the noradrenergic brain system. However, a translation of this stimulation technique to clinical practice is lacking. We therefore investigated the potential of vagal stimulation to inhibit excessive fear responses and facilitate responding to in-vivo and laboratory exposure in individuals with specific phobia. Spider-phobic participants were subjected to three standardized in-vivo exposures towards a living tarantula, complemented by an exposure in vitro (between exposure in vivo I and II). Transcutaneous auricular vagus nerve stimulation (taVNS) was applied during in-vitro exposure, presenting pictures of the exposed tarantula, other spiders and neutral tools in the laboratory. Fear was assessed by self-reports and behavioral avoidance (in-vivo exposures), and amygdala-mediated autonomic and behavioral fear components (exposure in vitro). Vagal stimulation facilitated the reduction of behavioral avoidance across repeated in-vivo exposures. During laboratory exposure, taVNS inhibited fear tachycardia and corrugator muscle activity specifically in response to pictures of the previously exposed tarantula – an effect that became stronger with increasing stimulation duration. Psychophysiological indices of noradrenergic transmission in the basolateral amygdala were elevated during taVNS and correlated to subsequent attenuation of behavioral avoidance. Our results suggest, that taVNS exerts stimulus-specific and dose-dependent inhibition of multiple automatic response components of excessive fear, highlighting taVNS as a valuable adjunct to exposure-based treatment. A translational mechanism of action is supported, proposing that taVNS exhibits its effects by noradrenergic activation of fear extinction circuitry, particularly targeting the basolateral amygdala.

## Introduction

Excessive fear and associated avoidance of perceived threat cues represent cardinal symptoms of anxiety disorders, constituting one of the largest groups of mental disorders and a leading cause of sustained disability [1–3]. The repeated exposure to fear-eliciting stimuli invokes a profound inhibition of these symptoms and has thus become a central strategy in anxiety disorder’s first-line treatment, cognitive behavioral therapy (CBT) [1, 4, 5]. Despite its efficacy, CBT nonetheless faces high rates of non-responders (~50%) and relapses (~14%), calling for therapeutic adjuncts that support a persistent inhibition of fear [6–8].

Translational research, which focuses on the underlying mechanisms of exposure treatment, provides valuable guidance for the development of such adjuncts. For instance, when rodents are repeatedly exposed to a previously threat-signaling stimulus that is now no longer associated with aversive consequences (extinction), fear responses decline due to increased transmission in the basolateral amygdala, which invokes an inhibition of the central amygdala that orchestrates multi-level fear responses [9, 10]. Such fear extinction is considered to be the core mechanism of exposure-based treatments [4, 8, 11, 12], and can even persist over longer time periods, if the medial prefrontal cortex activates the basolateral amygdala during future encounters of the previous threat cue [13, 14]. Preclinical functional imaging research suggested, that the human amygdala and medial prefrontal cortex exhibit similar functions during fear extinction as in rodents [12, 15–18]. Accordingly, a technique that taps into these neural circuits might be a promising adjunct to facilitate responding to exposure-based treatments.

Rodent research indicated, that the stimulation of the peripheral vagus nerve increases noradrenergic transmission in the basolateral amygdala via its projections to the locus coeruleus in the brainstem [19]. Furthermore, vagus nerve stimulation strengthens the connectivity between the basolateral amygdala and medial prefrontal cortex and thus in fact promotes extinction of even excessive fear [20–24]. Preclinical fear research in humans has largely replicated these findings using non-invasive transcutaneous auricular vagus nerve stimulation (taVNS), which also elevates noradrenergic signaling [25–27] and increases activity in the amygdala and prefrontal cortex [28, 29]. Most importantly, though, taVNS also promotes the extinction of threat expectancy, as well as physiological and behavioral indices of conditioned fear in a long-term manner and additionally attenuates return of fear phenomena in healthy humans [30–33]. Based on these findings, taVNS was rendered a promising potential candidate for an adjunct to CBT of anxiety disorders, possibly promoting treatment response and long-term persistence of fear inhibition. However, the efficacy of taVNS in the context of real-world therapeutic settings has not been tested so far.

**Fig. 1:**
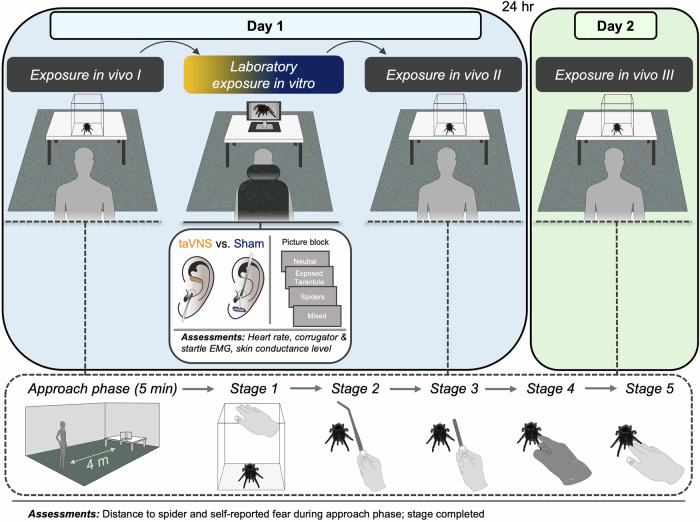
Schematic representation of the study protocol. On day 1, participants first underwent an exposure in vivo (exposure in vivo I), guided by a trained therapist. Here, participants first approached a tarantula inside a glass enclosure as closely as tolerable for 5 min, after which they were asked to interact with the tarantula in a hierarchical way: Touch the glass enclosure containing the tarantula (stage 1), touch the tarantula using long tongs (stage 2), a short pen (stage 3), while wearing gloves (stage 4) and finally with bare hands (stage 5). Distance to spider and fear level (during approach phase), as well as the completed stages were assessed. An exposure in vitro in the laboratory followed, during which four blocks of pictures were presented that included neutral tools (1), the exposed tarantula (2), other spiders (3) and both other spiders and the exposed tarantula (4; mixed block). Only during the laboratory session, taVNS vs. sham stimulation was applied by an experimenter, while heart rate, facial muscle activity and skin conductance was recorded. Further exposure in vivo sessions followed immediately after (exposure in vivo II) and 24 h later (exposure in vivo III; day 2). Across all study phases the participant and therapist were double-blinded to the stimulation type that had been applied in the laboratory.

We therefore applied taVNS to spider-phobic individuals and tested this potential during repeated threat exposures in a sham-controlled, double-blinded between-group design. Across two experimental days, participants were subjected to a total of three exposure in vivo sessions, during which they approached and interacted with a living tarantula. These in-vivo exposures were complemented by a laboratory exposure in vitro (between exposure in vivo I and II), during which participants received either active taVNS or a sham stimulation while viewing pictures of the previously exposed tarantula, other spiders and neutral tools – a strategy, shown to invoke an inhibition on the brain’s defense networks and subject’s fear responses similar to in-vivo exposures [34–37] (for details, see Fig. 1 and Methods and Materials). We assessed excessive fear responses on multiple levels, including reported feelings and behavioral avoidance during in-vivo exposures. During the laboratory exposure, we also assessed autonomic and indirect reflexive behavioral read-outs of amygdala activity, including changes in heart rate, corrugator muscle activity, skin conductance and startle reflex [38–44]. Importantly, these responses also provide insights about the actual defense state of the organism: Cardiac acceleration (*fear tachycardia*) signals a state of circa-strike defense, that is evoked when a threat is perceived as highly imminent and during which the organism is beyond vigilance and prepares for defensive action [41, 45–48]. Such state may further be accompanied by increases in skin conductance and corrugator muscle activity, that serve as objective indicators of elevated sympathetic arousal and negative affect, respectively [43, 44, 49]. By combining different forms of exposures, we therefore are not only able to examine, whether taVNS impacts on a persistent response to in-vivo exposure as conducted in CBT, but can also assess how taVNS modulates more automatic indices of the fear responses in a controlled laboratory environment (see Experimental design).

Based on previous research, showing that taVNS facilitates neural transmission in fear-inhibitory brain areas and correspondingly improves long-term fear inhibition [28–33], we hypothesized that taVNS would facilitate responding to repeated in-vivo exposures to a living spider in phobic individuals, as indexed by a promoted reduction of self-reported fear and behavioral avoidance. Furthermore, we expected taVNS to also inhibit fear expression on different response levels (i.e., heart rate, corrugator muscle activity, skin conductance and startle reflex) to visual threat cues during the laboratory exposure.

## Material and methods

### Participants

Thirty-three women who reported a strong fear of spiders were recruited from a student sample of the University of Greifswald. One subject was excluded from final analyses due to equipment failure, resulting in a final sample of 32 participants (*M*_*Age*_ = 22.4; *SD*_*Age*_ = 3.5; *Range* = 18–33; all right-handed). Based on an a-priori power analysis [50, 51] and previous work that indicated large effect sizes by taVNS on the inhibition of fear [31, 32], this sample size was sufficiently powered (i.e., ≥ 0.80) [52] to detect large effects throughout exposures in vitro and in vivo^1^. Furthermore, this sample size is also comparable to previous studies, that tested neuroscience-based adjuncts to exposure therapy [54].

Before study participation, each subject completed the German version of the Spider Phobia Questionnaire (SPQ; [55, 56]). Only subjects were invited to the study, who scored one standard deviation or greater above the population mean of the SPQ-15 (i.e., 4.07 ± 3.73). The SPQ-15 contains a subset of fifteen SPQ items with high face validity, that demonstrated to predict avoidance behavior and fear towards spiders in nonclinical and treatment-seeking samples, while also differentiating between spider-fearful vs. non-fearful and spider-phobic vs. non-phobic individuals [57]. In the included sample, the average SPQ-15 sum-score (*M*_*SPQ-15*_ = 10.91, *SD*_*SPQ-15*_ = 1.47, *Range* = 8–14) was comparable to sum-scores reported by spider-phobic patients in previous research (10.67 ± 1.79) [57]. We found no difference between the taVNS and sham group with regard to thus assessed fear of spiders (*F*_*Stimulation*_(1,31) = 0.127, *p* = 0.724). Aside from one participant, all subjects further reported that fear is being evoked as soon as they encounter a spider, that spiders are frequently avoided, that fear has been present for more than six months, and that impairments or burden result from their fear of spiders, indicative of a diagnosis of specific phobia [58].

Further inclusion criteria comprised an age between 18–35 years and a body-mass-index in normal range (18.5–27 kg/m^2^). Previous or current medical or mental conditions, that would have affected any of the outcome measures or would have conflicted with vagal stimulation led to an exclusion. This included any previous or current neurological, cardiovascular or other bodily condition (e.g., diabetes, neurodermatitis on the hand’s palm, hormonal disorders, impaired vision or hearing), the wearing of implants (e.g., pacemakers, cochlea implants), pregnancy, but also any history of psychotherapeutic treatment, as well as the previous or current use of psychotropic drugs. The applicability of in- and exclusion criteria was assessed on the basis of participants’ self-report. Participants received either course credits or financial compensation for study participation.

### Experimental design

In our study, taVNS was applied in a two-day sham-controlled between-group design, which comprised a total of three therapist-guided exposure in vivo sessions towards a tarantula, of which the first two (exposure in vivo I and II) took place on the first experimental day, while the third session (exposure in vivo III) followed 24 h later (Fig. 1).

Importantly, the first two exposure in vivo sessions were separated by a laboratory exposure in vitro, which was conducted by an independent experimenter and during which the participants were exposed to pictures of the previously exposed tarantula, other spiders and neutral stimuli (for details on the exposure procedures see Exposure in vivo and Laboratory exposure in vitro). Only during this laboratory exposure in vitro session, half of the participants received either active taVNS (*n* = 16) or a sham stimulation to the earlobe (*n* = 16), whereby the allocation to the respective stimulation condition was randomly determined beforehand. No stimulation was applied during the exposure in vivo sessions, ensuring that the stimulation condition was double-blinded to both participant and the therapist (for further details see Transcutaneous auricular vagus nerve stimulation). This design therefore allowed us to not only investigate the effects of taVNS on physiological and behavioral fear responses in a laboratory environment (exposure in vitro), but also to evaluate a taVNS-paired complementary exposure as a therapeutic adjunct, that might improve both immediate (exposure in vivo II) and persisting responding to treatment (exposure in vivo III).

### Exposure in vivo

The exposure in vivo sessions were conducted in a bright, elongated room, in which a glass enclosure containing the tarantula was placed on a table at a distance of 4 meters from the participant and therapist (see lower panel of Fig. 1). The tarantula was a specimen of *Tliltocatl albopilosus* – a rather docile, slow and easy to control species of tarantulas (leg span approximately 14 cm). Each exposure in vivo session started with a five-minute approach phase, during which the therapist asked the participant to approach the glass enclosure containing the tarantula as closely as tolerable, always starting at the maximum distance of 4 meters.

Immediately following the approach phase, participants were encouraged to accomplish different stages in a standardized behavioral approach task: Touch the glass enclosure (stage 1) containing the tarantula, touch the tarantula using long tongs (stage 2), touch the tarantula using a short pen (stage 3), touch the tarantula while wearing gloves (stage 4) and finally touch the tarantula with bare hands (5). Each stage was first demonstrated by the therapist and the participant could choose to repeat the respective interaction with the tarantula or to terminate the respective exposure session. To make sure that such approach behavior was reliable, a stage had only been regarded as accomplished if the respective interaction (e.g., touch the glass enclosure) was repeated three times. Only after a stage had been accomplished, the next stage was demonstrated and participants had the option to proceed with the behavioral approach task or not.

### Laboratory exposure in vitro

The laboratory exposure in vitro directly followed the first exposure in vivo (exposure in vivo I). During the laboratory exposure in vitro, subjects sat in a sound-attenuated and dimly-lit chamber, 1.45 m in front of a 27-inch computer screen (2560 × 1440 pixel resolution; see Fig. 1). After attachment of the electrodes for recording physiological signals (see Assessments and data reduction), the experimenter applied the electrodes for taVNS vs. sham stimulation according to the designated stimulation condition. To ensure sufficient vagus nerve activation, a stimulation intensity-workup followed, during which the amperage of the stimulation was individually adjusted for each participant to be clearly perceptible, but without painful discomfort (see Transcutaneous auricular vagus nerve stimulation). After a following startle habituation period, during which six acoustic startle probes (50 ms white noise; 95 dB(A); rise/fall time <1 ms) were presented to adapt participants’ startle magnitudes to a stable baseline, the laboratory exposure in vitro began. Here, a series of 48 color pictures was presented, that depicted neutral tools, the previously exposed tarantula and other spiders^2^. The presentation of visual stimuli was organized in four blocks of sixteen pictures each and pseudorandomized in four orders (counterbalanced across participants): [59, 60] The *Neutral* block only contained different pictures of neutral tools, while the *Exposed Tarantula* block only contained different pictures of the exposed tarantula from the exposure in-vivo sessions. The *Spiders* block only contained different pictures of other spiders than the exposed tarantula and the *Mixed* block contained both different pictures of the exposed tarantula (*n* = 8) and other spiders (*n* = 8). Each picture was presented on a grey background for a duration of 7.5 s via the monitor. After each picture, an inter-trial interval (ITI) followed, during which only the grey background was presented (10, 12 or 14 s; mean duration = 12 s). To elicit the startle eyeblink, startle probes were administered in 75% of the picture presentations (i.e., 12 startle probes for each block; 4.5, 5 or 6 s after picture onset, *M*_*Onset*_ = 5 s) and in 25% of the ITIs (i.e., 4 startle probes for each block; 5, 6, 7 or 8 s after ITI onset, *M*_*Onset*_ = 6.5 s). After the laboratory exposure in vitro was finished, all electrodes were removed and the participant proceeded with exposure in vivo II.

In this laboratory exposure session, the *Neutral* block served as a control condition to differentiate general defensive alertness of the participant from spider-related fear responses that are elicited in the remaining three blocks. Of the three blocks containing fear-related stimuli, the *Exposed Tarantula* block was used to probe stimulus-specific fear, as semantic/conceptual fear responses. In this block, semantically similar, but perceptually different fear stimuli were presented (comparable to fear generalization tests in basic research [61, 62]). Finally, the *Mixed* block was used to probe both stimulus-specific and higher order semantic fear responses, providing a condition in which participants could not anticipate the type of spider picture being presented. This design therefore allowed not only a detailed disentanglement of stimulus-specific vs. higher order semantic fear responses to spiders, but more importantly a thorough examination of the impact of taVNS on these different instances of fear.

### Transcutaneous auricular vagus nerve stimulation

Only during the laboratory exposure in vitro, transcutaneous auricular vagus nerve stimulation was delivered by a battery-driven constant current stimulator (CMO2, Cerbomed, Erlangen Germany) via two titanium electrodes, that were located at the left ear. In the taVNS condition, electrodes were placed in the cymba conchae – an area that is exclusively innervated by the left auricular branch of the vagus nerve [63]. In the sham condition, however, electrodes were placed in the center of the earlobe – an area that is only innervated by the great auricular nerve and not by any vagal afferents [63]. The stimulation intensity was individually adjusted for each participant to be perceptible, but not painful. To this end, participants were asked to manually ramp up the stimulation intensity in steps of 0.1 mA each, starting at an amperage of 0.1 mA. After each adjustment, they were required to rate their subjective sensation of the stimulation on a visual 11-point scale, ranging from “nothing felt” (0), “light tingling” (3), “strong tingling” (6) to “painful” (10). The intensity workup proceeded until an intensity was found, that was rated as “8” (i.e., strong tingling sensation without being painful) or if the maximum intensity that could be delivered by the stimulation device (i.e., 5.0 mA) was reached [31, 32]. The average stimulation intensity did not differ between the taVNS and sham condition (*M*_*taVNS*_ = 2.16, *M*_*Sham*_ = 3.10; *z* = −1.736, *p* = 0.086). The stimulation was applied throughout the entire exposure in vitro at a frequency of 25 Hz, with a pulse width of 200–300 µs and a 30 s ON-30 s OFF duty cycle. Importantly, stimulation electrodes were removed immediately after exposure in vitro, ensuring that only the experimenter, but neither the participant nor the therapist, was aware of the actually applied stimulation type. Hence, the exposure in vitro was single-blinded, whereas all exposure in vivo sessions were double-blinded. In line with recent research [64, 65], taVNS was well-tolerated and no adverse effects have been reported by the participants. However, side effects of stimulation have not been systematically investigated in the current study.

### Assessments and data reduction

#### Distance to spider and self-reported fear (exposure in vivo)

At the beginning of each exposure in vivo (I, II, and III), i.e., right after entering the room with the tarantula, baseline measures of the participant’s distance to the spider (always 4 m) and self-reported fear were assessed, closely adapted to previous research [66]. For the following 5 min of the approach phase, the distance to the spider in centimeters and self-reported fear (measured on a scale ranging from 0–10, with 0 representing “no fear” and 10 representing “very severe fear”) was assessed on a minute-by-minute basis. In sum, six distance and six fear scores have thus been obtained during each exposure in vivo (baseline and after 1, 2, 3, 4 and 5 min). Finally, the closest approach distance to the spider, as well as mean and maximum self-reported fear for each exposure session was calculated based on these scores.

#### Approach stages accomplished (exposure in vivo)

Following the approach phase of each exposure in vivo, a standardized behavioral approach task was conducted (see Exposure in vivo). Based on the last accomplished stage, the performance of the participant was rated from 0 to 5: 0 = not able to touch the glass enclosure, 1 = touched the glass enclosure containing the tarantula, 2 = touched the tarantula using long tongs, 3 = touched the tarantula using a short pen, 4 = touched the tarantula while wearing gloves, 5 = touched the tarantula with bare hands. Participants were finally considered as exposure completers, if they were able to touch the tarantula with bare hands (i.e., complete the fifth stage), while participants, who prematurely terminated the session, were considered as non-completers.

#### Heart rate (exposure in vitro)

Heart rate was measured by two electrolyte filled (Marquette Hellige, Freiburg, Germany) Ag/AgCl electrodes (8 mm diameter), that were applied using an Einthoven Lead II setup for electrocardiography (ECG). The raw ECG signal was amplified by a Coulbourn V75-04 isolated bioamplifier (Coulbourn Instruments, Holliston, MA, USA) and sampled at 2000 Hz by a Biopac MP160 system (Biopac, Goleta, CA, USA). Offline artifact correction was conducted using ANSLAB (v. 2.4; Autonomic Nervous System Laboratory, University of Basel, Switzerland). The signal was subsequently down-sampled at 400 Hz and filtered with a 8–13 Hz band-pass filter. After artifact correction, the ECG data was converted to heart rate in beats per minute for every half-second of the sampling period [67]. Based on the obtained scores, mean heart rate in beats per minute was computed for the entire laboratory exposure in vitro. More importantly, though, to quantify baseline-independent cardiac responding as a central outcome of the current study, heart rate during picture presentation was subtracted from a base period heart rate (mean of the first two half-seconds before picture onset) for every half-second of the stimulus presentation (i.e., 15 data points for the 7.5 s duration of the picture). Finally, the resulting half-second based difference scores (Δ bpm) were averaged across all trials of a picture block (*Neutral*, *Exposed Tarantula*, *Spiders*, *Mixed*) to obtain a baseline-independent grand average for cardiac change.

#### Corrugator muscle activity (exposure in vitro)

Corrugator muscle activity was measured by two electrolyte filled (Marquette Hellige, Freiburg, Germany) Ag/AgCl miniature surface electrodes (3 mm diameter, Sensormedics, Yorba Linda, CA, USA) that were placed above the left eye according to the recommendations by Fridlund and Cacioppo [68]. The EMG signal was amplified by a Coulbourn V75-04 isolated bioamplifier and sampled at 2000 Hz by a Biopac MP160 system. Next, the EMG data was down-sampled to 1000 Hz, filtered by a 30 Hz high-pass filter as well as smoothed and rectified with a time constant of 10 ms. Afterwards, corrugator EMG was converted to microvolts for every half-second of the sampling period [34, 69]. Based on the obtained scores, mean corrugator activity in microvolt was computed for the entire laboratory exposure in vitro. As for heart rate, baseline-independent corrugator activity change was quantified by subtracting corrugator activity during picture presentation from base period corrugator activity for every half-second of the stimulus presentation. Finally, the resulting half-second based difference scores (∆ µV) were averaged across all trials of a picture block (*Neutral*, *Exposed Tarantula*, *Spiders*, *Mixed*) to obtain a baseline-independent grand average for corrugator muscle activity change.

#### Startle eyeblink response (exposure in vitro)

The eyeblink component of the startle reflex, elicited by the acoustic startle probe, was measured by two electrolyte filled (Marquette Hellige, Freiburg, Germany) Ag/AgCl miniature surface electrodes (3 mm diameter, Sensormedic, Yorba Linda, CA, USA), which recorded the electromyographic activity of the orbicularis oculi muscle underneath the left eye. The EMG signal was amplified by a Coulbourn V75-04 isolated bioamplifier and sampled at 2000 Hz by a Biopac MP160 system. For the offline scoring of the startle eyeblinks, the EMG data was down-sampled to 1000 Hz, filtered by a 50 Hz notch-, a 60 Hz high-pass and 400 Hz low-pass filter, as well as smoothed and rectified with a time constant of 10 ms (see also 48). A custom computer program [70] was then used to detect startle eyeblink responses semi-automatically between 100 ms before and 400 ms after the startle probe onset. All detected startle responses were visually inspected for artifacts and manually corrected if necessary. Following previously published guidelines [71], startle responses were finally scored, if they started between 20–120 ms and peaked within 150 ms after the startle probe administration with a minimum amplitude of 1.954 μV. Trials were scored as zero responses if no blink was detected (*M*_*Zero*_: 2.5%). Trials with clear stimulation artifacts, movement artifacts or excessive baseline activity were set as missings (*M*_*Missing*_: 30.7%) [71]. After the scoring procedure, raw startle blink magnitudes were averaged for the habituation phase, ITIs and each picture block (*Neutral*, *Exposed Tarantula*, *Spiders*, *Mixed*) to compensate for missing values and obtain average startle magnitudes for each experimental condition.

#### Skin conductance level (exposure in vitro)

The skin conductance was measured at the participant’s non-dominant hand by two electrolyte filled (0.05 M sodium chloride) Ag/AgCl electrodes (8 mm diameter), which were attached on the hypothenar eminence of the palmar surface. A Coulbourn V71-23 skin conductance coupler, providing a constant current of 0.5 V across the two electrodes, amplified the signal, which was sampled at 2000 Hz by a Biopac MP160 system. Next, the skin conductance data was digitally sampled at a rate of 10 Hz and processed with a resolution of 0.001 µS. Based on the obtained scores, mean skin conductance level in microsiemens was computed for the entire laboratory exposure in vitro. As for heart rate and corrugator muscle activity, baseline-independent skin conductance level change was quantified by subtracting skin conductance during picture presentation from base period skin conductance for every half-second of the stimulus presentation. Finally, the resulting half-second based difference scores (∆ µS) were averaged across all trials of a picture block (*Neutral*, *Exposed Tarantula*, *Spiders*, *Mixed*) to obtain a baseline-independent grand average for skin conductance level change.

### Statistical analysis and figure creation

Linear mixed effect models were used to analyze the effect of *Stimulation* (taVNS vs. sham stimulation) on changes in distance towards the tarantula and self-reported fear across the three exposure in vivo *Sessions* (exposure in vivo I, II and III). Furthermore taking into account individual differences between participants, that might contribute to systematic variability in approach and fear responses (e.g., some participants might experience higher levels of fear on average than others), we also modeled a random intercept for each *Participant*. Likewise, the accomplishment of stages during the behavioral approach task was analyzed using ordinal mixed regression models that used *Stimulation* and *Session* as fixed effects and *Participant* as random intercept. Finally, to test whether taVNS affected the likelihood to fully complete the exposure in vivo (i.e., touch spider with bare hands), a logistic mixed effect regression was performed using *Stimulation* and *Session* as fixed effects and *Participant* as random intercept.

Linear mixed models were also used to analyze the fixed effect of *Stimulation* on fear responses during the laboratory exposure in vitro. To analyze the course of heart rate, corrugator muscle and skin conductance activity change, averaged for each picture *Block* (*Neutral*, *Exposed Tarantula*, *Spiders*, *Mixed*), these models additionally included the fixed effect of *Half Second* (1–15 half-second bins during picture presentation). Again, *Participant* was included as a random intercept in these models. Similarly, averaged raw magnitudes were analyzed using *Stimulation* and *Block* (also including the averaged ITI startle magnitudes) as fixed effects, while *Participant* served as random intercept. As taVNS effects might be dose-dependent and thus vary over time, we additionally conducted analyses testing whether stimulation effects varied during between the first and second *Half* (first 8 trials vs. second 8 trials) of a respective block.

Mixed effect models were chosen over traditional analysis strategies like standard regression, as they use all available data for analysis, while standard regression typically excludes participants upon missing values, hereby compromising statistical power [72]. Second, mixed effect models also account for systematic interindividual differences between participants by including random effects, while in standard regression residual variance is solely interpreted as error variance [73].

Finally, we also wanted to test whether the hypothesized taVNS effects on fear-related measures during the laboratory exposure in vitro would be linked to the performance in the following exposure in vivo sessions (exposure in vivo II and III). To this end, we computed the correlation between fear indices during the laboratory exposure in vitro (e.g., mean startle magnitude), and fear indices during exposure in vivo II and III (e.g., the percentage of exposure completers).

Analyses were performed using *R* [74]. Linear, ordinal and logistic mixed effect models were created using the *lmer* function of the *lmerTest* package [75], the *clmm2* function of the *ordinal* package [76], and the *glmer* function of the *lme4* package [77], respectively. The significance of fixed effects in linear mixed models was tested by *F*-tests using the *anova* function of the *stats* package [74]. For logistic mixed effect models, the significance of effects was tested by Wald χ^2^-tests using the *Anova* function of the *car* package [78]. Pearson correlations were computed using the *cor.test* function in *R* [74]. The level of statistical significance was set to *p* < 0.05. Figures were created with help of Microsoft PowerPoint, Excel, Adobe Illustrator and https://app.displayr.com.

## Results

### Vagus nerve stimulation facilitates responding to repeated exposures in vivo

**Fig. 2:**
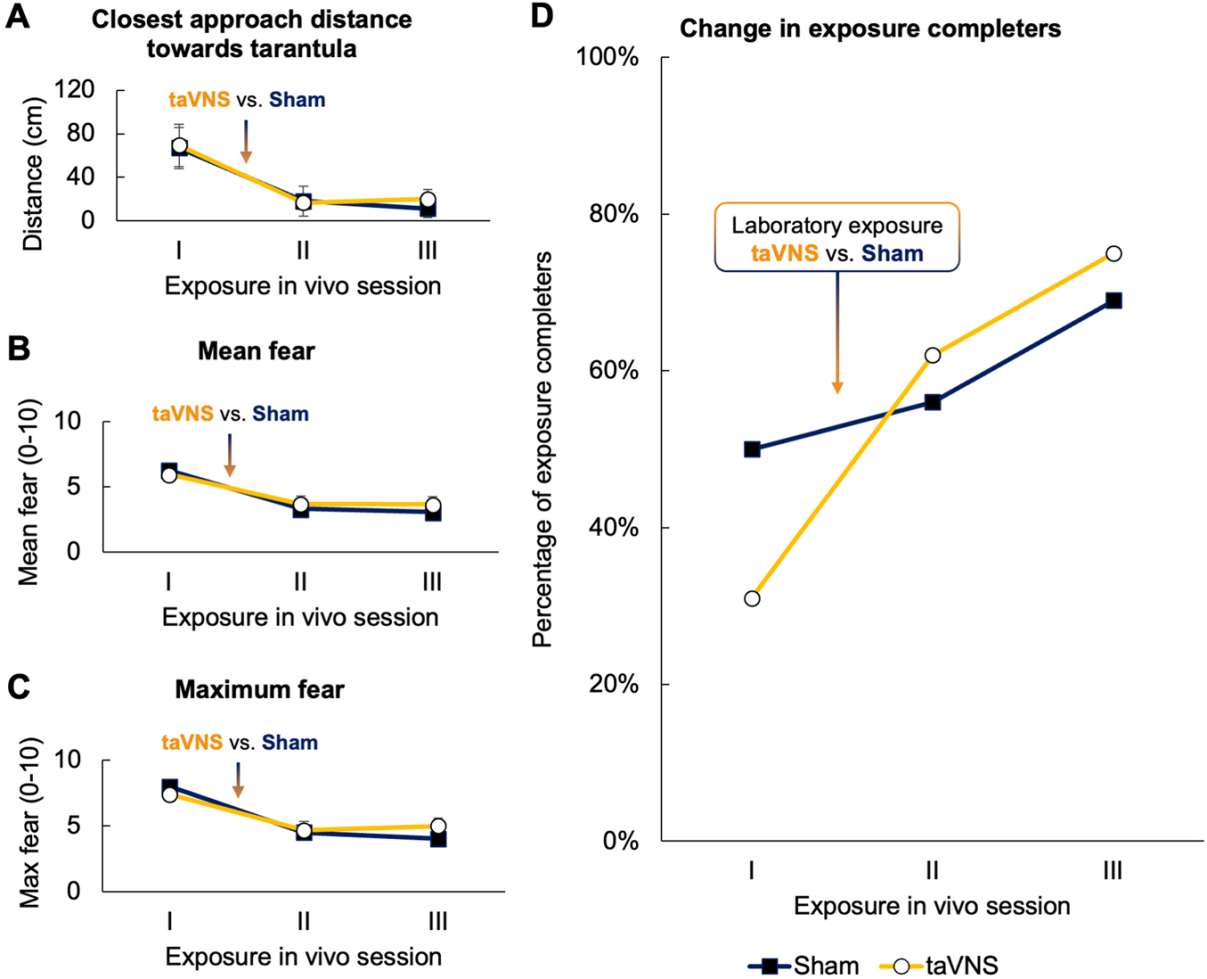
Vagus nerve stimulation facilitates responding to repeated in vivo exposures. Change in approach distance towards the tarantula (**A**), mean (**B**) and maximum self-reported fear (0–10; **C**), and percentage of exposure completers (i.e., participants that accomplished every stage during the behavioral approach task; **D**) across the three exposure in vivo sessions for the sham (blue lines) and taVNS condition (orange lines). Error bars indicate the standard error of the mean.

Across the three exposure in vivo sessions, participants’ fear responses successively declined: With increasing number of exposures to the tarantula, participants were able to approach the phobic stimulus more closely (*Session*, *F*_1,62_ = 25.267, *p* < 0.001; Fig. 2A), but also reported less overall fear (*Session*, *F*_1,62_ = 50.422, *p* < 0.001; Fig. 2B) as well as maximum fear (*Session*, *F*_1,62_ = 51.737, *p* < 0.001; Fig. 2C). Similarly, participants were able to accomplish more stages during the behavioral approach task across the three sessions (*Session*, *β* = 3.103, *p* = 0.003; see Supplemental Fig. 1), and thus the likelihood of fully completing the exposure, i.e., touching the tarantula with bare hands, also increased significantly (*Session*, ${{\rm{\chi }}}_{1}^{2}$ = 18.711, *p* < 0.001; change from 40.6% full completers during session I to 71.9% completers during session III; Fig. 2D). The results therefore indicate that the repeated exposure sessions were effective at reducing both subjective and behavioral indices of fear activation in phobic participants.

**Fig. 3:**
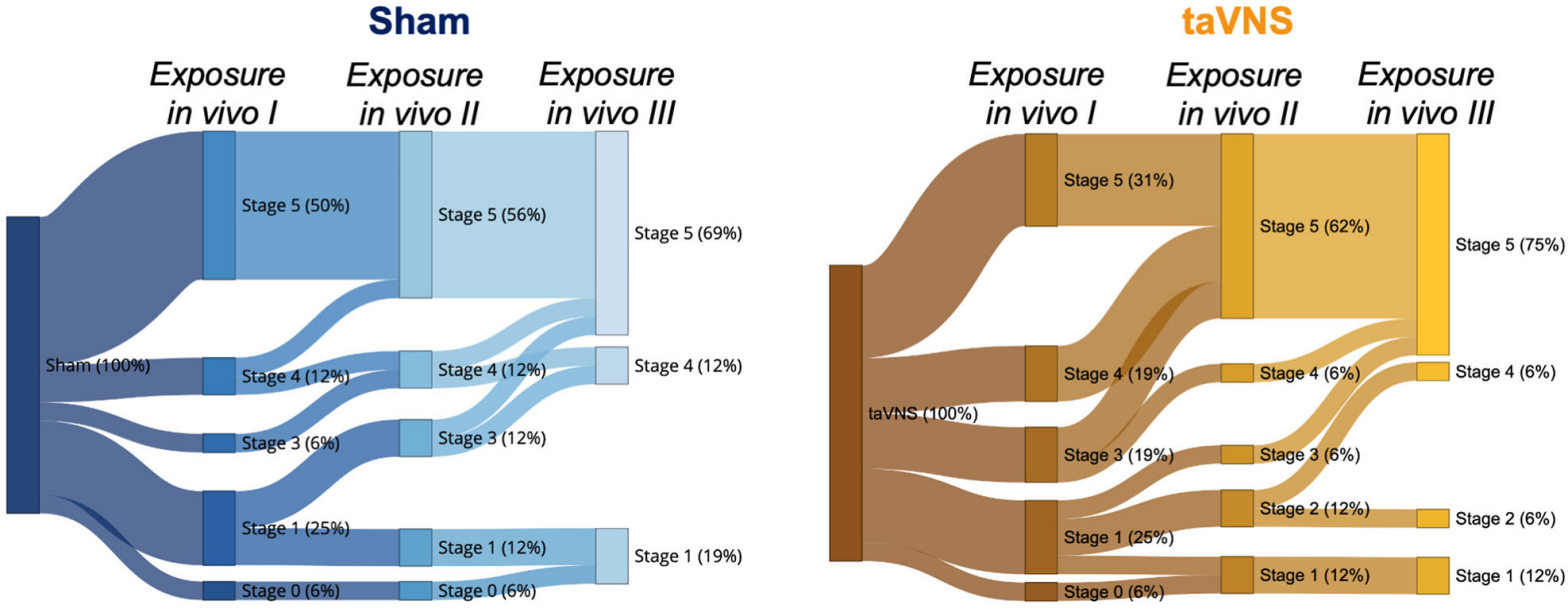
Vagus nerve stimulation increases the likelihood to fully complete repeated exposures. Sankey plot showing the flow of participants of the sham (left panel) and taVNS condition (right panel) at accomplishing different stages across the three exposure in vivo sessions.

Critically, taVNS significantly facilitated the reduction of fear responses across the three exposure sessions, albeit very selectively: Vagal stimulation did not affect the change in distance to the spider or self-reported fear across the exposure sessions (*Session x Stimulation*, all *ps* ≥ 0.079; Fig. 2A–C). However, participants who underwent a taVNS-paired laboratory exposure (after exposure in vivo I, see Fig. 1) benefited more from the repeated exposure in vivo sessions with regard to their performance during the therapist-guided behavioral approach task: Although the interaction of *Stimulation* and *Session* was non-significant (*β* = 1.801, *p* = 0.116), we observed that the odds to accomplish more stages on the task were six times higher in the taVNS relative to the sham condition (*Odds Ratio* = 6.052; Supplemental Fig. 1). Accordingly, we observed that vagal stimulation significantly increased the probability of touching the tarantula and thus completing the exposure in vivo across the three sessions (*Session x Stimulation*, ${{\rm{\chi }}}_{1}^{2}$ = 3.944, *p* = 0.047; Fig. 2D, Fig. 3).

While the percentage of exposure completers only marginally increased from 50% before the stimulation was applied (exposure in vivo I) to 56% after the participants underwent the laboratory exposure in vitro under sham stimulation (exposure in vivo II), the percentage of completers doubled from 31% (exposure in vivo I) to 62% (exposure in vivo II) when participants received taVNS (Fig. 2D, Fig. 3). Importantly, these effects maintained 24 h later, as the percentage of completers further increased from 56% (exposure in vivo II) to 69% (exposure in vivo III) in the sham condition, while it increased from 62 to 75% in the taVNS condition (Fig. 2D, Fig. 3). These results suggest, that taVNS facilitated the rate, at which phobic individuals responded to exposure.

Critically, however, while there were no significant differences between the taVNS and sham group with regard to distance covered towards the tarantula, self-reported fear or accomplished stages during the behavioral approach task (all *ps* ≥ 0.431), we found that both groups already differed in the percentage of full completers before the stimulation was applied, i.e., during exposure in vivo I (see Fig. 2D). The taVNS group was less likely to *fully* complete the behavioral approach task (percentage of completers: 30%) compared to the sham condition (percentage of completers: 50%) during the first exposure in vivo (*p* < 0.001). To rule out that the above mentioned interaction effect was solely driven by different baseline response rates, we therefore tested the effect of vagal stimulation on the likelihood to fully complete the exposure in vivo only for session II and III (i.e., after stimulation administration), while controlling for the performance in the first exposure in vivo as a dichotomous covariate (completer vs. non-completer). This analysis revealed a significant main effect of *Stimulation* (${{\rm{\chi }}}_{1}^{2}$ = 8.220, *p* = 0.004), indicating that participants who received taVNS during the laboratory exposure were five times more likely to fully complete the exposure in vivo during session II and III as compared to the sham condition (*Odds Ratio*: 5.474). Most importantly, this effect was independent of the participants’ performance during the first behavioral approach task (exposure in vivo I; *Completer/Non-Completer x Stimulation*, ${{\rm{\chi }}}_{1}^{2}$ = 0.011, *p* = 0.917), suggesting that taVNS exhibited its fear-inhibiting effects regardless of the performance during the preceding exposure sessions.

### Vagus nerve stimulation facilitates the inhibition of stimulus-specific fear during the laboratory exposure in vitro

**Fig. 4:**
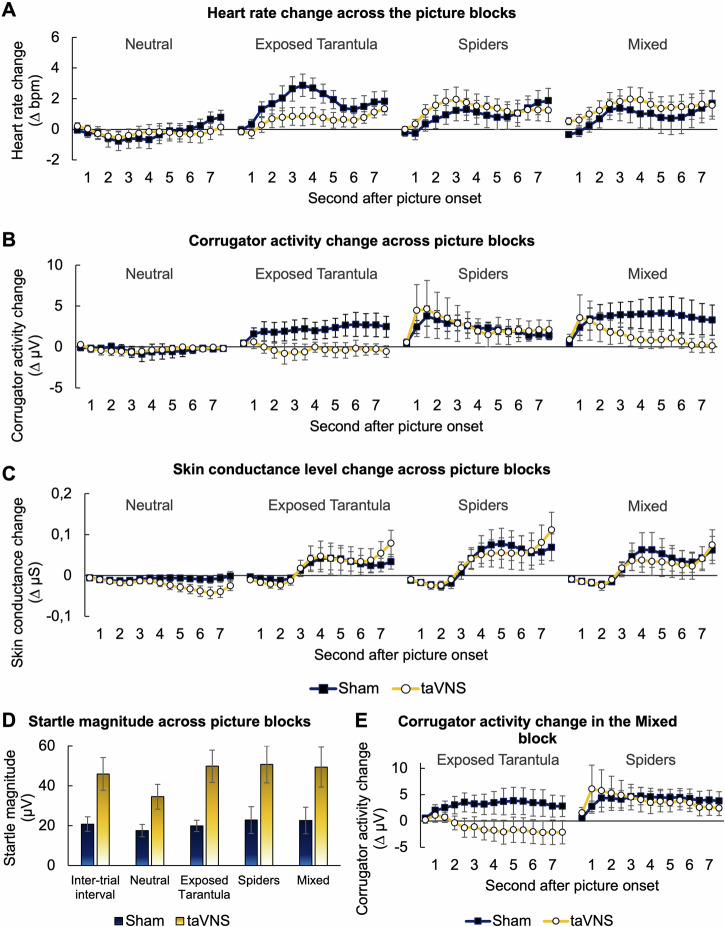
Vagus nerve stimulation inhibits physiological and behavioral indices of excessive fear. Averaged change in heart rate (**A**), corrugator muscle activity (**B**) and skin conductance level (**C**) for each half-second of the picture presentation and each of the four experimental blocks. **D** Averaged startle magnitudes elicited during the inter-trial interval (ITI) and the four experimental blocks. **E** Corrugator activity change (half-second bins) for pictures of the *Mixed* block that depicted the exposed tarantula (left) or other spiders (right). Blue lines/bars represent data of the sham condition, whereas orange lines/bars represent data of the taVNS condition. Error bars represent the standard error of the mean.

Importantly, fear-reducing effects were not only found after taVNS, but also during stimulation in the laboratory exposure in vitro (i.e., between exposure in vivo I and II). Here, we observed that pictures of the *Exposed Tarantula* and *Spiders*, presented in non-mixed or mixed blocks, elicited significant increases in heart rate (*Block*, *F*_3,1770_ = 51.549, *p* < 0.001; Fig. 4A), corrugator muscle activity (*Block*, *F*_3,1770_ = 59.940, *p* < 0.001; Fig. 4B), skin conductance level (*Block*, *F*_3,1770_ = 37.033, *p* < 0.001; Fig. 4C) as well as startle reflex magnitudes (*Block*, *F*_4,120_ = 2.977, *p* = 0.022; Fig. 4D) as compared to the *Neutral* block. These results indicate that pictures of any spider elicited substantial fear in our phobic participants.

Critically, these indices of fear activation were specifically attenuated by taVNS: While we did not find a difference in overall cardiac responding between the taVNS and sham condition (*Stimulation*, *F*_1,30_ = 0.006, *p* = 0.939; see also Supplemental Results), taVNS inhibited heart rate increase specifically in response to the pictures of the exposed tarantula (*Exposed Tarantula* block, *Block x Stimulation*, *F*_3,1770_ = 15.064, *p* < 0.001; Fig. 4A). Similarly, while there was no overall effect of stimulation (*Stimulation*, *F*_1,30_ = 0.799, *p* = 0.379), taVNS also inhibited the increase in corrugator activity, again, particularly in the *Exposed Tarantula* block as compared to a sham stimulation (*Block x Stimulation*, *F*_3,1770_ = 17.994, *p* < 0.001; Fig. 4B). In addition, we also observed reduced corrugator muscle activity in the *Mixed* block, when participants received taVNS relative to sham stimulation (*Block x Stimulation*, *F*_3,1770_ = 17.994, *p* < 0.001; Fig. 4B). However, a closer examination revealed, that the attenuating effect of taVNS on corrugator activity was again specific for the pictures showing the previously exposed tarantula (*Half Second x Stimulation*, *F*_14,420_ = 2.413, *p* = 0.002; Fig. 4E). No such taVNS effect was observed for pictures of other spiders (*Half Second x Stimulation*, *F*_14,420_ = 0.818, *p* = 0.649; Fig. 4E). Thus, the results indicate that taVNS inhibits behavioral and physiological indices of stimulus-specific fear, but not higher order semantic fear responses, suggesting a taVNS-specific effect on fear extinction rather than fear generalization.

**Fig. 5:**
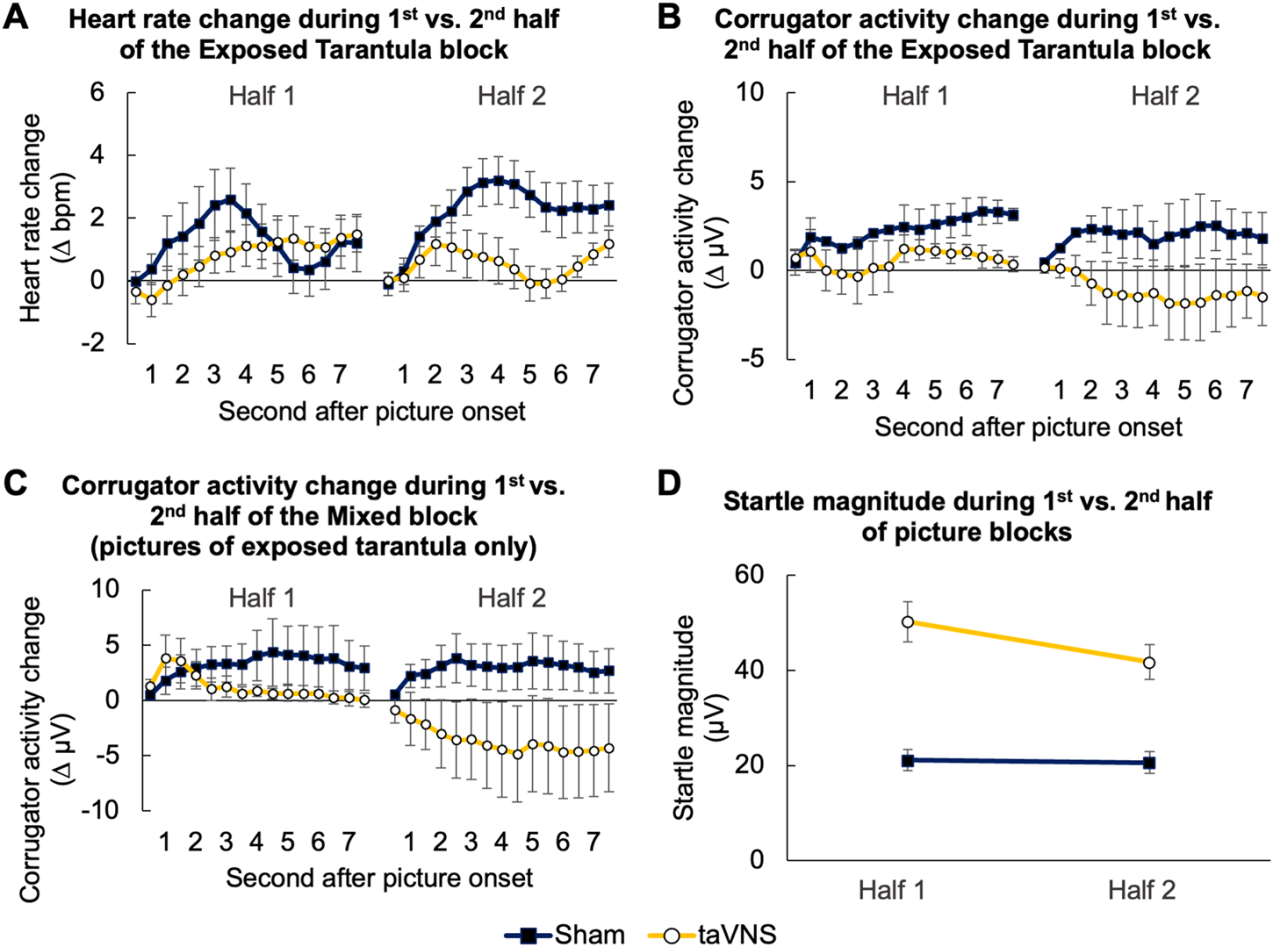
Effects of vagal stimulation change with increasing stimulation duration. Averaged change in heart rate (**A**) and corrugator muscle activity (**B**) during the first and second half of pictures that were presented in the *Exposed Tarantula* block. **C** Averaged change in corrugator muscle activity during the first and second half of pictures depicting the exposed tarantula that were presented in the *Mixed* block. **D** Change in startle magnitudes elicited during the first and second half of a picture block (averaged across all blocks). Blue lines represent data of the sham condition, whereas orange lines represent data of the taVNS condition. Error bars represent the standard error of the mean.

Interestingly, the fear-reducing effect of taVNS became stronger with increasing stimulation duration: The reduction in heart rate and corrugator muscle activity by taVNS was significantly more pronounced during the second half of the *Exposed Tarantula* picture block relative to the first one (*Half x Stimulation*, heart rate: *F*_1,870_ = 15.775, *p* < 0.001; corrugator: *F*_1,870_ = 8.579, *p* = 0.003; Fig. 5A, B). This pattern was also found for corrugator responses towards pictures of the exposed tarantula in the *Mixed* block, which were more strongly attenuated by taVNS during the second relative to the first half of picture presentation (*Half x Stimulation*, *F*_14,870_ = 28.338, *p* < 0.001; Fig. 5C). The results therefore indicate that fear inhibition by taVNS was more pronounced the longer the stimulation was administered.

No differences were found between the taVNS and sham condition with regard to changes in the skin conductance level (all *ps* ≥ 0.228; Fig. 4C). We also did not observe that taVNS (compared to sham) modulated startle magnitudes differently for particular picture blocks or the ITI (*Block x Stimulation*, *F*_4,120_ = 0.999, *p* = 0.411; Fig. 4D). However, we did observe a higher overall startle magnitudes in the taVNS relative to the sham condition throughout all blocks as well as the inter-trial intervals (*Stimulation*, *F*_1,30_ = 8.521, *p* = 0.007; Fig. 4D), which was also found for habituation startles (*p* = 0.012, see also Supplemental Results), indicating that taVNS generally sensitized startle magnitudes irrespective of the presented visual stimuli (see Supplemental Fig. 2). Despite such sensitization, mean startle magnitudes declined the longer taVNS was applied, while they remained on the same level in the sham condition (*Half x Stimulation*, *F*_1,269.017_ = 5.272, *p* = 0.022; Fig. 5D), suggesting that vagal stimulation promotes an adaptation to aversive stimuli (i.e., the startle probe) the longer the stimulation was applied.

### Vagus nerve stimulation facilitates the attenuation of independent fear expressions

Interestingly, correlational analysis indicated that the fear responses elicited by the pictured vs. real exposed tarantula, which were attenuated by vagal stimulation, were also primarily unrelated. Whether the participants fully completed the behavioral approach task and touched the tarantula with bare hands during exposure in vivo II or III was not related to any of the physiological and behavioral responses towards the pictures of the exposed tarantula, on which taVNS had a significant effect in the laboratory – neither overall (all *ps* ≥ 0.209) nor separately in the sham (all *ps* ≥ 0.059) and taVNS condition (all *ps* ≥ 0.114; see Supplemental Table 1) Thus, the results suggest that vagal stimulation exhibited attenuating effects on multiple, but independent components of stimulus-specific fear activation in both exposure settings.

**Fig. 6:**
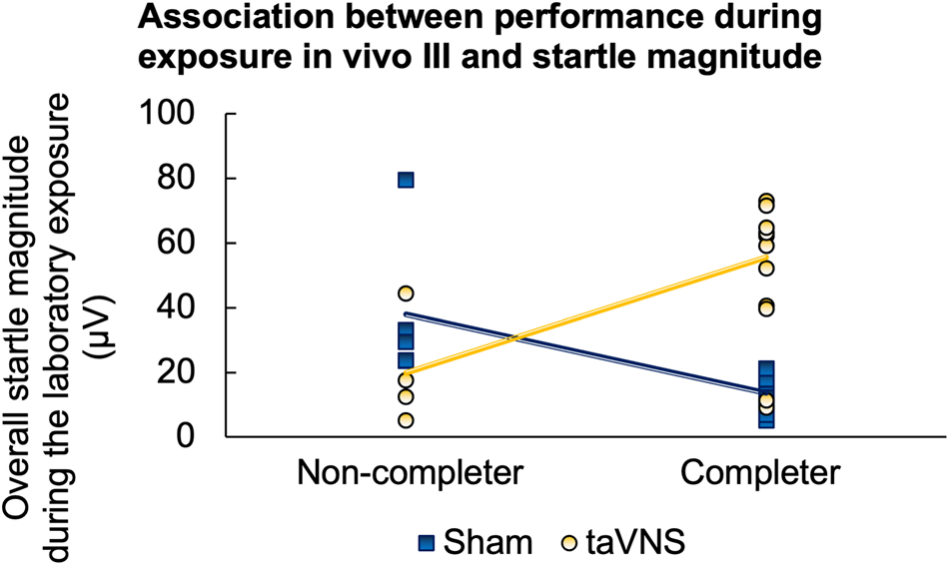
Sensitized startle reflexes are associated with responding to in-vivo exposure. Scatter plot for overall startle magnitudes elicited during the laboratory exposure in participants that completed (right) or did not complete the exposure in vivo III. Dots and lines represent data and point-biserial correlation for the sham (blue) and taVNS condition (orange), respectively.

For sensitized startle magnitudes, however, we observed a different pattern: When calculating the correlation separately for each stimulation condition, we found that higher startle reflex magnitudes positively predicted the eventual completion of exposure in vivo III in the taVNS condition (*r* = 0.745, *p* < 0.001), while the opposite was the case if participants received sham stimulation (*r* = −0.667, *p* = 0.005, see Fig. 6 and also Supplemental Table 1). These results indicate that during laboratory exposure taVNS generally increased the (sensory) processing of the aversive startle probes, which was also positively linked to successful approach in the in-vivo exposures.

## Discussion

Vagus nerve stimulation has been rendered a potential adjunct to exposure treatments, due to its capacity to promote the extinction of fear in the face of previous threat signals in laboratory studies [20–23, 30–33, 79]. We tested this translational potential, for the first time, in the context of a real-world therapeutic setting in spider-phobic individuals. To this end, we applied non-invasive transcutaneous auricular vagus nerve stimulation (taVNS) during a laboratory exposure towards phobic stimuli (spider pictures), that was conducted between three in-vivo exposures towards a living tarantula (between the first two in-vivo exposures). In line with our hypothesis, we observed that behavioral avoidance towards the living tarantula markedly declined after participants underwent the taVNS-paired laboratory exposure, which was maintained 24 h later. Specifically, it was more likely to make physical contact with the phobic stimulus at similar levels of self-reported fear and approach distance in taVNS vs. sham-stimulated subjects. However, attenuated fear activation not only unfolded after, but was already evident *while* taVNS was administered: During the laboratory exposure, taVNS significantly inhibited fear tachycardia and corrugator muscle activity – autonomic and behavioral fear components that serve as indirect read-outs of amygdala activity [41–44]. Such inhibition was more pronounced the longer the stimulation was applied, indicating a dose-dependency of taVNS effects. However, taVNS effects only emerged, if pictures of the actually exposed tarantula, but not pictures of other spiders, were presented. Furthermore, fear-attenuating effects during laboratory and in-vivo exposures were not related to each other. Our data therefore suggest that taVNS invokes a stimulus-specific inhibition of multiple and partly independent fear components, that however does not generalize to semantically similar, yet perceptually different fear cues. Most importantly, though, our results strongly indicate that taVNS may be utilized to enhance the efficacy of exposure-based treatment by promoting the reduction of behavioral avoidance.

In rodents, the basolateral amygdala (BLA) is the critical site of fear extinction [10] – a stimulus-specific memory process, by which fear responses towards a distinct cue are inhibited, if it loses its attribute as a threat signal [11, 80–82]. The BLA organizes this process, as it evaluates a cue with regard to its threat-signaling value and increasingly inhibits fear responses organized by the central amygdala upon repeated non-reinforced exposures [9, 10]. Invasive vagal stimulation may facilitate this plastic fear extinction process by increasing noradrenergic (NA) transmission and long-term potentiation in the basolateral amygdala, possibly via vagal projections to the brainstem locus coeruleus [19, 24, 83]. Non-invasive taVNS may engage the same mechanism by increasing NA-dependent activation of the BLA, which leads to facilitated processing of a distinct cue that loses its threat-signaling value, ultimately resulting in inhibited amygdala-dependent fear components.

In fact, preclinical human research provides partial support for this view, as it showed that taVNS increases locus-coeruleus-dependent NA-transmission, enhances NA-dependent memory formation for salient stimuli, and elevates the blood flow in the amygdala [25, 29, 84, 85]. Our study extends this picture and provides further evidence, that taVNS increases noradrenergic signaling in the BLA: Rodent research rendered startle sensitization a marker of increased NA transmission in the BLA after the exposure to significant threats [86]. Specifically, after rats are exposed to a predator (a ferret), stress increases the responsiveness of NA receptors in the BLA and thus subsequent noradrenaline infusions may invoke BLA sensitization, that manifests as heightened startle [86]. In the current study, the same pattern was observed after participants had been exposed to a potent fear cue (the tarantula) followed by the administration of taVNS, possibly indicating that vagal stimulation invoked a similar NA-kindling of the BLA after threat exposure. Consistent with animal research, showing that such increased noradrenergic transmission in the BLA promotes fear inhibition [83], startle sensitization positively predicted whether the participants of the taVNS condition eventually made physical contact with the tarantula.

However, heightened startle reflexes may not only reflect increased NA-signaling after threat exposure, but also increased motivated attention [31, 87], possibly suggesting that taVNS facilitated the processing of emotional cues. In line with this view, previous research showed that taVNS enhances the P3b, but also emotion discrimination as reflected in the LPP– event-related potentials associated with elaborated stimulus evaluation and sustained attention, respectively [27, 85, 88]. In our study, heart rate data provides further support, that taVNS induced a state of facilitated attentional processing: Sham-stimulated participants responded with a profound cardiac acceleration towards pictures of the exposed tarantula. Such fear tachycardia has been interpreted as a hallmark of circa-strike defense – a highly aversive defense state elicited by perceived imminent threat, during which the organism is beyond vigilance and prepares for defensive action (e.g., avoidance) [41, 45, 47, 89–91]. Accordingly, fear tachycardia has also been interpreted as an index of sensory rejection [92, 93]. Such fear tachycardia was markedly attenuated when participants received taVNS, which may suggest that vagal stimulation alleviates perceived threat imminence and thus helps the organism to revoke from circa-strike defense, ultimately setting the organism in a state that allows a more thorough processing of fear cues. Accordingly, taVNS also reduced corrugator muscle activity, possibly reflecting reduced negative affect which is usually dominant during circa-strike defense [90, 94].

As the attenuating effects of vagal stimulation particularly targeted fear responses, that are controlled by the amygdala (fear tachycardia, corrugator muscle activity, behavioral avoidance [41–44, 95]), our study provides further evidence that taVNS indeed inhibited the amygdala output. Conscious feelings of fear, however, are presumed to be less dependent on amygdala signaling, but to be organized by a broad assembly of cortical areas [96, 97]. A reduction of self-reported fear across the repeated in-vivo exposures was not further promoted by taVNS. Our data therefore suggest that taVNS has not fully targeted this assembly and possibly also indicate, that taVNS inhibits maladaptive amygdala-dependent defensive responses rather than cognitive indices of fear activation. Alternatively, this pattern of results might be grounded in the efficacy of in-vivo exposures: In our study, fear ratings decreased by nearly 50% across the three exposure in-vivo sessions, which may represent a floor effect that did not allow a further promotion by taVNS.

Finally, the fear-attenuating effects of taVNS maintained 24 h after the stimulation was applied. This is consistent with previous literature, showing that vagal stimulation activates projection targets of the locus coeruleus noradrenergic system, not only including subcortical amygdala-circuits [19], but also the medial prefrontal cortex (mPFC) [20] – an area, shown to critically mediate the consolidation and retention of fear extinction memory [12, 15, 98]. Our data therefore indicate that taVNS facilitated fear inhibition by tapping into the BLA, and consolidated fear-inhibition by tapping into the mPFC, both mediated by increased locus-coeruleus-dependent NA-transmission [20, 24, 98]. In line with previous research that showed promoted long-term fear inhibition by taVNS for 24 h and even 4 weeks [32], this may suggest that the potential long-term effects of vagus nerve stimulation could also be helpful in reducing relapses, which should be further investigated in future studies.

Taken together, we found evidence that taVNS inhibits stimulus-specific amygdala-dependent fear responses on multiple levels of expression in spider phobic individuals, likely driven by central noradrenergic activation of neural fear extinction circuits. Thus, the current study not only refines our concept of the mechanisms underlying taVNS, but also allows a critical evaluation of its capacity to inhibit excessive fear responses, finally highlighting its potential use as an adjunct to exposure therapy. In fact, exposure-based treatment of anxiety disorders majorly builds upon extinction processes [4, 8, 11, 12] and deficient fear extinction is prevalent in patients with anxiety disorders [99], possibly contributing to high rates of non-responders and relapses [6, 7]. Extending previous evidence [20–24, 30–33], our results suggest that taVNS may help to overcome these deficits by noradrenergically tapping into fear extinction circuitry, ultimately facilitating responding to such treatment. Hence, taVNS might yield important advantages in comparison to other methods that aim to facilitate CBT, such as pharmacological interventions that target extinction circuits via administration of glucocorticoids or NMDAR-agonist D-cycloserine (DCS) [100–104]. In contrast to these substances, which exhibit effects by acting upon broadly distributed brain networks, vagal stimulation constitutes a technique to non-invasively activate a fairly circumscribed circuitry (i.e., the vagal afferent network), particularly hinged upon locus-coeruleus-dependent NA-transmission [105]. Our data therefore indicate, that vagal stimulation may yield a similar potential as glucocorticoids and DCS to tap into extinction circuits and thus facilitate the attenuation of even excessive fear, but stands out for taking a specific noradrenergic route to achieve these effects – notably with high safety and tolerability [64, 65].

It is important to mention, however, that the effects of vagal stimulation therefore are specific in two ways: First, and consistent with the notion that vagal stimulation might primarily tap into amygdala-dependent extinction circuits, the current study suggests taVNS may only promote a sustained reduction of behavioral avoidance, while cognitive indices of fear (e.g., the feeling of fear) remain unaffected. Second, and consistent with findings that fear extinction is a stimulus-specific process [80–82], the current study indicates that taVNS promotes a rather stimulus-specific attenuation of fear indices, while higher order conceptual/generalized fear (e.g., towards spiders in general) may not be influenced. This is also in line with the assumption that taVNS exerts its effects via the central noradrenaline system, as previous findings showed that noradrenaline facilitates memory accuracy rather than memory generalization, which is more dependent on glucocorticoid transmission [100, 101, 106]. In sum, taVNS may be considered an effective adjunct to facilitate and maintain responding to exposure-based treatment of anxiety disorders, but additional strategies seem necessary to further reduce cognitive fear indices and evoke a generalization of fear inhibition to conceptually related fear cues.

## Limitations

Although the current results indicate, that taVNS may enhance the efficacy of exposure-based therapy, there may be some features that could limit a full generalization to clinical practice: (1) The current sample included only female participants who also shared a similar social status (university students). To render taVNS as a universal tool to enhance CBT, future research therefore needs to test, whether this stimulation technique also facilitates exposure effects in larger and more diverse samples. (2) taVNS promoted responding to exposure for spider phobic participants; it may, however, not be warranted that similar effects occur for other anxiety disorders, that are marked by more generalized anxious apprehension (e.g., generalized anxiety disorder [58]). In fact, there is evidence that taVNS may not reduce negative thought intrusions during worry induction [107]. To define broader areas of clinical application, future research need to test whether taVNS may promote exposure for different types of mental disorders, possibly also including obsessive-compulsive, stressor- and trauma-related disorders. (3) While repeated in-vivo exposures have been found to effectively evoke self-reported fear reduction, taVNS did not further promote such effect in our study. Additional strategies therefore seem necessary to optimize the treatment success on the level of verbal reports, e.g., by adjusting stimulation parameters (e.g., intensity) in a way that maximizes the induced neuroplasticity [108] or by applying complementary therapeutic techniques, such as focusing on a change in threat expectancy [109]. (4) In the current study, vagal stimulation was only applied during a complementary laboratory exposure, but never during actual in-vivo exposure sessions. This approach was chosen, as it is the only way to comprehensively measure stimulus-specific and higher-order fear responses using psychophysiological methods, while also ensuring that the therapist and participant are double-blinded to the stimulation condition (otherwise trained therapists would have identified active taVNS and sham stimulation based on different electrode positions). A further important reason for this approach was that taVNS has shown to promote memory for aversive emotional experiences [85]. Accordingly, we wanted to avoid that potential adverse events, that are more likely to occur during less-controllable in-vivo exposures (e.g., a panic attack), may be particularly consolidated by taVNS. Encouraged by the current promising results, future research should however test whether taVNS exhibits similar fear-attenuating effects as in the current study if applied during in-vivo exposures. (5) Future studies should also include some variability in exposure contexts in order to test whether taVNS leads to context-independent fear attenuation: In fact, the current study kept the context of in-vivo exposures identical across the three different sessions. However, fear renewal due to contextual switches is a common phenomenon in both basic research and clinical practice [66, 110, 111]. While animal research has provided evidence that vagus nerve stimulation also attenuates fear renewal [112], this effect has yet to be proven in humans.

## Supplementary information

Supplemental Material

## Data Availability

Data that was used in this study, is available upon request from the corresponding author.

## Footnotes

1 In the current study, we used (generalized) linear mixed regression approaches for data analyses (see Statistical analysis). Since a-priori (i.e., non-data-based) power simulations with specified effect sizes are difficult for mixed models, as regression coefficients cannot be easily related to effect sizes [53], the described power analysis was run for less-powered fixed-effect regression models, thus providing a lower bound of actually achieved statistical power.

2 The pictures of neutral tools and other spiders were mainly selected from the International Affective Picture System [59]. The IAPS identification numbers for the neutral pictures were: 7000, 7002, 7003, 7004, 7006, 7009, 7010, 7016, 7017, 7018, 7021, 7025, 7034, 7035, 7043, 7045, 7046, 7050, 7055, 7056, 7059, 7080, 7090, 7095, 7150, 7175, 7185, 7190, 7211, 7233, 7235, 7236. The identification numbers for the spider pictures were 1200, 1202, 1205, 1230, 1240. Further spider pictures were selected from picture sets that were successfully utilized in previous studies [60].

## Code availability

Code that was used in this study, is available upon request from the corresponding author.

## References

[1] Craske MG, Stein MB, Eley TC, Milad MR, Holmes A, Rapee RM, et al. Anxiety disorders. Nat Rev Dis Primers. 2017;3:17024. 28470168 10.1038/nrdp.2017.24 PMC11009418

[2] Baxter AJ, Vos T, Scott KM, Ferrari AJ, Whiteford HA. The global burden of anxiety disorders in 2010. Psychol Med. 2014;44:2363–74. 24451993 10.1017/S0033291713003243

[3] Baxter AJ, Scott KM, Vos T, Whiteford HA. Global prevalence of anxiety disorders: A systematic review and meta-regression. Psychol Med. 2013;43:897–910. 22781489 10.1017/S003329171200147X

[4] Craske MG, Treanor M, Conway CC, Zbozinek T, Vervliet B. Maximizing exposure therapy: An inhibitory learning approach. Behav Res Ther. 2014;58:10–23. 24864005 10.1016/j.brat.2014.04.006 PMC4114726

[5] Carpenter JK, Andrews LA, Witcraft SM, Powers MB, Smits JAJ, Hofmann SG. Cognitive behavioral therapy for anxiety and related disorders: A meta‐analysis of randomized placebo‐controlled trials. Depress Anxiety. 2018;35:502–14. 29451967 10.1002/da.22728 PMC5992015

[6] Levy HC, O’Bryan EM, Tolin DF. A meta-analysis of relapse rates in cognitive-behavioral therapy for anxiety disorders. J Anxiety Disord. 2021;81:102407. 33915506 10.1016/j.janxdis.2021.102407

[7] Loerinc AG, Meuret AE, Twohig MP, Rosenfield D, Bluett EJ, Craske MG. Response rates for CBT for anxiety disorders: Need for standardized criteria. Clin Psychol Rev. 2015;42:72–82. 26319194 10.1016/j.cpr.2015.08.004

[8] Fanselow MS. Fear and anxiety take a double hit from vagal nerve stimulation. Biol Psychiatry. 2013;73:1043–4. 23647735 10.1016/j.biopsych.2013.03.025 PMC4176918

[9] Herry C, Ciocchi S, Senn V, Demmou L, Müller C, Lüthi A. Switching on and off fear by distinct neuronal circuits. Nature. 2008;454:600–6. 18615015 10.1038/nature07166

[10] Tovote P, Fadok JP, Lüthi A. Neuronal circuits for fear and anxiety. Nat Rev Neurosci. 2015;16:317–31. 25991441 10.1038/nrn3945

[11] Dunsmoor JE, Niv Y, Daw N, Phelps EA. Rethinking extinction. Neuron. 2015;88:47–63. 26447572 10.1016/j.neuron.2015.09.028 PMC4598943

[12] Milad MR, Quirk GJ. Fear extinction as a model for translational neuroscience: ten years of progress. Annu Rev Psychol. 2012;63:129–51. 22129456 10.1146/annurev.psych.121208.131631 PMC4942586

[13] Senn V, Wolff SBE, Herry C, Grenier F, Ehrlich I, Gründemann J, et al. Long-range connectivity defines behavioral specificity of amygdala neurons. Neuron. 2014;81:428–37. 24462103 10.1016/j.neuron.2013.11.006

[14] Burgos-Robles A, Vidal-Gonzalez I, Santini E, Quirk GJ. Consolidation of fear extinction requires NMDA receptor-dependent bursting in the ventromedial prefrontal cortex. Neuron. 2007;53:871–80. 17359921 10.1016/j.neuron.2007.02.021

[15] Szeska C, Pünjer H, Riemann S, Meinzer M, Hamm AO. Stimulation of the ventromedial prefrontal cortex blocks the return of subcortically mediated fear responses. Transl Psychiatry. 2022;12:394. 36127327 10.1038/s41398-022-02174-8 PMC9489865

[16] Phelps EA, Delgado MR, Nearing KI, LeDoux JE. Extinction learning in humans: Role of the amygdala and vmPFC. Neuron. 2004;43:897–905. 15363399 10.1016/j.neuron.2004.08.042

[17] Keller NE, Hennings AC, Leiker EK, Lewis-Peacock JA, Dunsmoor JE. Rewarded extinction increases amygdalar connectivity and stabilizes long-term memory traces in the vmPFC. J Neurosci. 2022;42:5717–29. 35680411 10.1523/JNEUROSCI.0075-22.2022 PMC9302464

[18] Raij T, Nummenmaa A, Marin MF, Porter D, Furtak S, Setsompop K, et al. Prefrontal cortex stimulation enhances fear extinction memory in humans. Biol Psychiatry. 2018;84:129–37. 29246436 10.1016/j.biopsych.2017.10.022 PMC5936658

[19] Hassert DL, Miyashita T, Williams CL. The effects of peripheral vagal nerve stimulation at a memory-modulating intensity on norepinephrine output in the basolateral amygdala. Behav Neurosci. 2004;118:79–88. 14979784 10.1037/0735-7044.118.1.79

[20] Peña DF, Childs JE, Willett S, Vital A, McIntyre CK, Kroener S. Vagus nerve stimulation enhances extinction of conditioned fear and modulates plasticity in the pathway from the ventromedial prefrontal cortex to the amygdala. Front Behav Neurosci. 2014;8:327. 25278857 10.3389/fnbeh.2014.00327 PMC4166996

[21] Peña DF, Engineer ND, McIntyre CK. Rapid remission of conditioned fear expression with extinction training paired with vagus nerve stimulation. Biol Psychiatry. 2013;73:1071–7. 23245749 10.1016/j.biopsych.2012.10.021 PMC3604026

[22] Noble LJ, Meruva VB, Hays SA, Rennaker RL, Kilgard MP, McIntyre CK. Vagus nerve stimulation promotes generalization of conditioned fear extinction and reduces anxiety in rats. Brain Stimul. 2019;12:9–18. 30287193 10.1016/j.brs.2018.09.013 PMC6301121

[23] Noble LJ, Gonzalez IJ, Meruva VB, Callahan KA, Belfort BD, Ramanathan KR, et al. Effects of vagus nerve stimulation on extinction of conditioned fear and post-traumatic stress disorder symptoms in rats. Transl Psychiatry. 2017;7:e1217. 28892066 10.1038/tp.2017.191 PMC5611754

[24] Alvarez-Dieppa AAC, Griffin K, Cavalier S, Mcintyre CK. Vagus nerve stimulation enhances extinction of conditioned fear in rats and modulates Arc protein, CaMKII, and GluN2B-containing NMDA receptors in the basolateral amygdala. Neural Plast. 2016;2016:1–19. 10.1155/2016/4273280 PMC5120198 27957346

[25] Wienke C, Grueschow M, Haghikia A, Zaehle T. Phasic, event-related transcutaneous auricular vagus nerve stimulation modifies behavioral, pupillary, and low-frequency oscillatory power responses. J Neurosci. 2023;43:6306–19. 37591736 10.1523/JNEUROSCI.0452-23.2023 PMC10490471

[26] Giraudier M, Ventura-Bort C, Burger AM, Claes N, D’Agostini M, Fischer R, et al. Evidence for a modulating effect of transcutaneous auricular vagus nerve stimulation (taVNS) on salivary alpha-amylase as indirect noradrenergic marker: a pooled mega-analysis. Brain Stimul. 2022;15:1378–88. 36183953 10.1016/j.brs.2022.09.009

[27] Ventura-Bort C, Wirkner J, Genheimer H, Wendt J, Hamm AO, Weymar M. Effects of transcutaneous vagus nerve stimulation (tVNS) on the P300 and alpha-amylase level: a pilot study. Front Hum Neurosci. 2018;12:202. 10.3389/fnhum.2018.00202. 29977196 10.3389/fnhum.2018.00202 PMC6021745

[28] Höper S, Kaess M, Koenig J. Prefrontal cortex oxygenation and autonomic nervous system activity under transcutaneous auricular vagus nerve stimulation in adolescents. Auton Neurosci. 2022;241:103008. 10.1016/j.autneu.2022.103008. 35724559 10.1016/j.autneu.2022.103008

[29] Frangos E, Ellrich J, Komisaruk BR. Non-invasive access to the vagus nerve central projections via electrical stimulation of the external ear: FMRI evidence in humans. Brain Stimul. 2015;8:624–36. 25573069 10.1016/j.brs.2014.11.018 PMC4458242

[30] Burger AM, Verkuil B, Van Diest I, Van der Does W, Thayer JF, Brosschot JF. The effects of transcutaneous vagus nerve stimulation on conditioned fear extinction in humans. Neurobiol Learn Mem. 2016;132:49–56. 27222436 10.1016/j.nlm.2016.05.007

[31] Szeska C, Richter J, Wendt J, Weymar M, Hamm AO. Attentive immobility in the face of inevitable distal threat—Startle potentiation and fear bradycardia as an index of emotion and attention. Psychophysiology. 2021;58:1–17. 10.1111/psyp.13812 33759212

[32] Szeska C, Richter J, Wendt J, Weymar M, Hamm AO. Promoting long-term inhibition of human fear responses by non-invasive transcutaneous vagus nerve stimulation during extinction training. Sci Rep. 2020;10:1529. 32001763 10.1038/s41598-020-58412-w PMC6992620

[33] Burger AM, Verkuil B, Fenlon H, Thijs L, Cools L, Miller HC, et al. Mixed evidence for the potential of non-invasive transcutaneous vagal nerve stimulation to improve the extinction and retention of fear. Behav Res Ther. 2017;97:64–74. 28719827 10.1016/j.brat.2017.07.005

[34] Wendt J, Lotze M, Weike AI, Hosten N, Hamm AO. Brain activation and defensive response mobilization during sustained exposure to phobia-related and other affective pictures in spider phobia. Psychophysiology. 2008;45:205–15. 17995911 10.1111/j.1469-8986.2007.00620.x

[35] Goossens L, Sunaert S, Peeters R, Griez EJL, Schruers KRJ. Amygdala hyperfunction in phobic fear normalizes after exposure. Biol Psychiatry. 2007;62:1119–25. 17706612 10.1016/j.biopsych.2007.04.024

[36] Siegel P, Cohen B, Warren R. Nothing to fear but fear itself: a mechanistic test of unconscious exposure. Biol Psychiatry. 2022;91:294–302. 34763847 10.1016/j.biopsych.2021.08.022

[37] Wendt J, Schmidt LE, Lotze M, Hamm AO. Mechanisms of change: effects of repetitive exposure to feared stimuli on the brain’s fear network. Psychophysiology. 2012;49:1319–29. 22913381 10.1111/j.1469-8986.2012.01451.x

[38] Kuhn M, Wendt J, Sjouwerman R, Büchel C, Hamm A, Lonsdorf TB. The neurofunctional basis of affective startle modulation in humans: evidence from combined facial electromyography and functional magnetic resonance imaging. Biol Psychiatry. 2020;87:548–58. 31547934 10.1016/j.biopsych.2019.07.028

[39] Davis M, Whalen PJ. The amygdala: vigilance and emotion. Mol Psychiatry. 2001;6:13–34. 11244481 10.1038/sj.mp.4000812

[40] Knight DC, Smith CN, Cheng DT, Stein EA, Helmstetter FJ. Amygdala and hippocampal activity during acquisition and extinction of human fear conditioning. Cogn Affect Behav Neurosci. 2004;4:317–25. 15535167 10.3758/cabn.4.3.317

[41] Roelofs K, Dayan P. Freezing revisited: coordinated autonomic and central optimization of threat coping. Nat Rev Neurosci. 2022;23:568–80. 35760906 10.1038/s41583-022-00608-2

[42] Lanteaume L, Khalfa S, Regis J, Marquis P, Chauvel P, Bartolomei F. Emotion induction after direct intracerebral stimulations of human amygdala. Cereb Cortex. 2007;17:1307–13. 16880223 10.1093/cercor/bhl041

[43] Heller AS, Lapate RC, Mayer KE, Davidson RJ. The face of negative affect: trial-by-trial corrugator responses to negative pictures are positively associated with amygdala and negatively associated with ventromedial prefrontal cortex activity. J Cogn Neurosci. 2014;26:2102–10. 24669790 10.1162/jocn_a_00622 PMC4117711

[44] Lee H, Heller AS, van Reekum CM, Nelson B, Davidson RJ. Amygdala–prefrontal coupling underlies individual differences in emotion regulation. Neuroimage. 2012;62:1575–81. 22634856 10.1016/j.neuroimage.2012.05.044 PMC3408571

[45] Löw A, Weymar M, Hamm AO. When threat is near, get out of here: dynamics of defensive behavior during freezing and active avoidance. Psychol Sci. 2015;26:1706–16. 26408036 10.1177/0956797615597332

[46] Wendt J, Löw A, Weymar M, Lotze M, Hamm AO. Active avoidance and attentive freezing in the face of approaching threat. Neuroimage. 2017;158:196–204. 28669911 10.1016/j.neuroimage.2017.06.054

[47] Hamm AO. Fear, anxiety, and their disorders from the perspective of psychophysiology. Psychophysiology. 2020;57:1–14. 10.1111/psyp.13474 31529522

[48] Signoret-Genest J, Schukraft N L, Reis S, Segebarth D, Deisseroth K, Tovote P. Integrated cardio-behavioral responses to threat define defensive states. Nat Neurosci. 2023;26:447–57. 36759559 10.1038/s41593-022-01252-w PMC9991919

[49] Lang PJ, GREENWALD MK, BRADLEY MM, Hamm AO. Looking at pictures: affective, facial, visceral, and behavioral reactions. Psychophysiology. 1993;30:261–73. 8497555 10.1111/j.1469-8986.1993.tb03352.x

[50] Faul F, Erdfelder E, Buchner A, Lang A-G. Statistical power analyses using G*Power 3.1: Tests for correlation and regression analyses. Behav Res Methods. 2009;41:1149–60. 19897823 10.3758/BRM.41.4.1149

[51] Faul F, Erdfelder E, Lang A-G, Buchner A. G*Power 3: A flexible statistical power analysis program for the social, behavioral, and biomedical sciences. Behav Res Methods. 2007;39:175–91. 17695343 10.3758/bf03193146

[52] Cohen J. A power primer. Psychol Bull. 1992;112:155–9. 19565683 10.1037//0033-2909.112.1.155

[53] Kumle L, Võ MLH, Draschkow D. Estimating power in (generalized) linear mixed models: an open introduction and tutorial in R. Behav Res Methods. 2021;53:2528–43. 33954914 10.3758/s13428-021-01546-0 PMC8613146

[54] Soeter M, Kindt M. An abrupt transformation of phobic behavior after a post-retrieval amnesic agent. Biol Psychiatry. 2015;78:880–6. 25980916 10.1016/j.biopsych.2015.04.006

[55] Klorman R, Weerts TC, Hastings JE, Mela BG, Lang PJ. Psychometric description of some specific-fear questionnaires 1 all rights of reproduction in any form reserved. Behav Ther. 1974;5:401–9.

[56] Hamm AO. Spezifische Phobien. Göttingen: Hogrefe; 2006.

[57] Olatunji BO, Woods CM, de Jong PJ, Teachman BA, Sawchuk CN, David B. Development and initial validation of an abbreviated spider phobia questionnaire using item response theory. Behav Ther. 2009;40:114–30. 19433143 10.1016/j.beth.2008.04.002

[58] American Psychiatric Association. *Diagnostic and statistical manual of mental disorders (5th ed.)*. Washington, DC: American Psychiatric Publishing, Inc. 2013. 10.1176/appi.books.9780890425596.

[59] Lang PJ, Bradley MM, Cuthbert BN. International affective picture system (IAPS): digitized photographs, instruction manual and affective ratings. 2005. 10.1037/t66667-000.

[60] Globisch J, Hamm AO, Esteves F, Öhman A. Fear appears fast: temporal course of startle reflex potentiation in animal fearful subjects. Psychophysiology. 1999;36:66–75. 10098381 10.1017/s0048577299970634

[61] Dunsmoor JE, Martin A, LaBar KS. Role of conceptual knowledge in learning and retention of conditioned fear. Biol Psychol. 2012;89:300–5. 22118937 10.1016/j.biopsycho.2011.11.002 PMC3269535

[62] Dunsmoor JE, Murphy GL. Categories, concepts, and conditioning: how humans generalize fear. Trends Cogn Sci. 2015;19:73–7. 25577706 10.1016/j.tics.2014.12.003 PMC4318701

[63] Peuker ET, Filler TJ. The nerve supply of the human auricle. Clin Anat. 2002;15:35–7. 11835542 10.1002/ca.1089

[64] Kim AY, Marduy A, de Melo PS, Gianlorenco AC, Kim CK, Choi H, et al. Safety of transcutaneous auricular vagus nerve stimulation (taVNS): a systematic review and meta-analysis. Sci Rep. 2022;12:1–16. 36543841 10.1038/s41598-022-25864-1 PMC9772204

[65] Giraudier M, Ventura-Bort C, Szeska C, Weymar M. A pooled analysis of the side effects of non-invasive transcutaneous auricular vagus nerve stimulation (taVNS). Front Hum Neurosci. 2025;19:1539416 10.3389/fnhum.2025.1539416. 39981126 10.3389/fnhum.2025.1539416 PMC11841445

[66] Mystkowski JL, Craske MG, Echiverri AM. Treatment context and return of fear in spider phobia. Behav Ther. 2002;33:399–416. 10.1016/j.beth.2005.04.001 16942960

[67] Graham FK. Constraints on measuring heart rate and period sequentially through real and cardiac time. Psychophysiology. 1978;15:492–5. 693763 10.1111/j.1469-8986.1978.tb01422.x

[68] Fridlund AJ, Cacioppo JT. Guidelines for human electromyographic research. Psychophysiology. 1986;23:567–89. 3809364 10.1111/j.1469-8986.1986.tb00676.x

[69] Klepzig K, Stender K, Lotze M, Hamm AO. Written in the face? facial expressions during pleasant and unpleasant chills. Psychol Music. 2023;51:952–70.

[70] Globisch J, Hamm AO, Schneider R, Vaitl D. A computer program for scoring reflex eyeblink and electrodermal responses written in PASCAL. Psychophysiology. 1993;30:30.8416060

[71] Blumenthal TD, Cuthbert BN, Filion DL, Hackley S, Lipp OV, Van Boxtel A. Committee report: guidelines for human startle eyeblink electromyographic studies. Psychophysiology. 2005;42:1–15. 15720576 10.1111/j.1469-8986.2005.00271.x

[72] Bagiella E, Sloan RP, Heitjan DF. Mixed-effects models in psychophysiology. Psychophysiology. 2000;37:13–20. 10705763

[73] Van Dongen HPA, Olofsen E, Dinges DF, Maislin G. Mixed-model regression analysis and dealing with interindividual differences. Essent Numer Comput Methods. 2004;384:139–71. 10.1016/S0076-6879(04)84010-2 15081686

[74] R Core Team. R: A language and environment for statistical computing. R Foundation for Statistical Computing. 2022. https://cran.r-project.org/.

[75] Kuznetsova A, Brockhoff PB, Christensen RHB. lmerTest package: tests in linear mixed effects models. J Stat Softw. 2017;82:1–26.

[76] Christensen RHB. Cumulative link models for ordinal regression with the R Package ordinal. 2018. 10.32614/CRAN.package.ordinal.

[77] Bates D, Mächler M, Bolker B, Walker S. Fitting linear mixed-effects models using lme4. J Stat Softw. 2015;67:1–48. 10.18637/jss.v067.i01.

[78] Fox J, Weisberg S *An R companion to applied regression*. 3rd ed. Thousand Oaks, CA: Sage, 2019.

[79] Noble LJ, Souza RR, McIntyre CK. Vagus nerve stimulation as a tool for enhancing extinction in exposure-based therapies. Psychopharmacology. 2019;236:355–67. 30091004 10.1007/s00213-018-4994-5 PMC6368475

[80] Vervoort E, Vervliet B, Bennett M, Baeyens F. Generalization of human fear acquisition and extinction within a novel arbitrary stimulus category. PLoS One. 2014;9:e96569. 24798047 10.1371/journal.pone.0096569 PMC4010469

[81] Hauner KK, Howard JD, Zelano C, Gottfried JA. Stimulus-specific enhancement of fear extinction during slow-wave sleep. Nat Neurosci. 2013;16:1553–5. 24056700 10.1038/nn.3527 PMC3818116

[82] Vervliet B, Vansteenwegen D, Eelen P. Generalization gradients for acquisition and extinction in human contingency learning. Exp Psychol. 2006;53:132–42. 16909938 10.1027/1618-3169.53.2.132

[83] Berlau DJ, McGaugh JL. Enhancement of extinction memory consolidation: The role of the noradrenergic and GABAergic systems within the basolateral amygdala. Neurobiol Learn Mem. 2006;86:123–32. 16458544 10.1016/j.nlm.2005.12.008

[84] Weymar M, Löw A, Modess C, Engel G, Gründling M, Petersmann A, et al. Propranolol selectively blocks the enhanced parietal old/new effect during long-term recollection of unpleasant pictures: a high density ERP study. Neuroimage. 2010;49:2800–6. 19837180 10.1016/j.neuroimage.2009.10.025

[85] Ventura-Bort C, Wirkner J, Wendt J, Hamm AO, Weymar M. Establishment of emotional memories is mediated by vagal nerve activation: evidence from noninvasive taVNS. J Neurosci. 2021;41:7636–48. 34281991 10.1523/JNEUROSCI.2329-20.2021 PMC8425981

[86] Rajbhandari AK, Baldo BA, Bakshi VP. Predator stress-induced CRF release causes enduring sensitization of basolateral amygdala norepinephrine systems that promote PTSD-Like startle abnormalities. J Neurosci. 2015;35:14270–85. 26490866 10.1523/JNEUROSCI.5080-14.2015 PMC4683687

[87] Lang PJ, Bradley MM, Cuthbert BN Motivated attention: affect, activation and action. In: Lang PJ, Simons RF, Balaban MT (eds). Attention and orienting: sensory and motivational processes. Hillsdale, NJ: Lawrence Erlbaum Associates, Inc.; 1997. pp. 97–135.

[88] Nelson BD, Hodges A, Hajcak G, Shankman SA. Anxiety sensitivity and the anticipation of predictable and unpredictable threat: Evidence from the startle response and event-related potentials. J Anxiety Disord. 2015;33:62–71. 26005838 10.1016/j.janxdis.2015.05.003 PMC4480216

[89] Lang PJ, Davis M. Emotion, motivation, and the brain: reflex foundations in animal and human research. Prog Brain Res. 2006;156:3–29. 17015072 10.1016/S0079-6123(06)56001-7

[90] Mobbs D, Marchant JL, Hassabis D, Seymour B, Tan G, Gray M, et al. From threat to fear: the neural organization of defensive fear systems in humans. J Neurosci. 2009;29:12236–43. 19793982 10.1523/JNEUROSCI.2378-09.2009 PMC2782300

[91] Fanselow MS. Neural organization of the defensive behavior system responsible for fear. Psychon Bull Rev. 1994;1:429–38. 24203551 10.3758/BF03210947

[92] Bradley MM. Natural selective attention: orienting and emotion. Psychophysiology. 2009;46:1–11. 18778317 10.1111/j.1469-8986.2008.00702.x PMC3645482

[93] Graham FK, Clifton RK. Heart-rate change as a component of the orienting response. Psychol Bull. 1966;65:305–20. 5325894 10.1037/h0023258

[94] Mobbs D, Headley DB, Ding W, Dayan P. Space, time, and fear: survival computations along defensive circuits. Trends Cogn Sci. 2020;24:228–41. 32029360 10.1016/j.tics.2019.12.016

[95] Weera MM, Shackett RS, Kramer HM, Middleton JW, Gilpin NW. Central amygdala projections to lateral hypothalamus mediate avoidance behavior in rats. J Neurosci. 2021;41:61–72. 33188067 10.1523/JNEUROSCI.0236-20.2020 PMC7786206

[96] LeDoux JE, Pine DS. Using neuroscience to help understand fear and anxiety: a two-system framework. Am J Psychiatry. 2016;173:1083–93. 27609244 10.1176/appi.ajp.2016.16030353

[97] LeDoux JE, Brown R. A higher-order theory of emotional consciousness. Proc Natl Acad Sci USA. 2017;114:E2016–E25. 28202735 10.1073/pnas.1619316114 PMC5347624

[98] Uematsu A, Tan BZ, Ycu EA, Cuevas JS, Koivumaa J, Junyent F, et al. Modular organization of the brainstem noradrenaline system coordinates opposing learning states. Nat Neurosci. 2017;20:1602–11. 28920933 10.1038/nn.4642

[99] Duits P, Cath DC, Lissek S, Hox JJ, Hamm AO, Engelhard IM, et al. Updated meta-analysis of classical fear conditioning in the anxiety disorders. Depress Anxiety. 2015;32:239–53. 25703487 10.1002/da.22353

[100] de Quervain DJ-F, Wolf OT, Roozendaal B. Glucocorticoid-induced enhancement of extinction—from animal models to clinical trials. Psychopharmacology. 2019;236:183–99. 30610352 10.1007/s00213-018-5116-0 PMC6373196

[101] de Quervain DJ-F, Bentz D, Michael T, Bolt OC, Wiederhold BK, Margraf J, et al. Glucocorticoids enhance extinction-based psychotherapy. Proc Natl Acad Sci. 2011;108:6621–5. 21444799 10.1073/pnas.1018214108 PMC3081033

[102] de Quervain DJ-F, Schwabe L, Roozendaal B. Stress, glucocorticoids and memory: implications for treating fear-related disorders. Nat Rev Neurosci. 2016;18:7–19. 27881856 10.1038/nrn.2016.155

[103] Otto MW, Kredlow MA, Smits JAJ, Hofmann SG, Tolin DF, de Kleine RA, et al. Enhancement of psychosocial treatment with D-cycloserine: models, moderators, and future directions. Biol Psychiatry. 2016;80:274–83. 26520240 10.1016/j.biopsych.2015.09.007 PMC4808479

[104] Davis M, Ressler K, Rothbaum BO, Richardson R. Effects of D-cycloserine on extinction: translation from preclinical to clinical work. Biol Psychiatry. 2006;60:369–75. 16919524 10.1016/j.biopsych.2006.03.084

[105] Hachem LD, Wong SM, Ibrahim GM. The vagus afferent network: emerging role in translational connectomics. Neurosurg Focus. 2018;45:E2. 30173606 10.3171/2018.6.FOCUS18216

[106] Bahtiyar S, Gulmez Karaca K, Henckens MJAG, Roozendaal B. Norepinephrine and glucocorticoid effects on the brain mechanisms underlying memory accuracy and generalization. Mol Cell Neurosci. 2020;108:103537. 32805389 10.1016/j.mcn.2020.103537

[107] Burger AM, Van der Does W, Thayer JF, Brosschot JF, Verkuil B. Transcutaneous vagus nerve stimulation reduces spontaneous but not induced negative thought intrusions in high worriers. Biol Psychol. 2019;142:80–9. 30710565 10.1016/j.biopsycho.2019.01.014

[108] Morrison RA, Hulsey DR, Adcock KS, Rennaker RL, Kilgard MP, Hays SA. Vagus nerve stimulation intensity influences motor cortex plasticity. Brain Stimul. 2019;12:256–62. 30409712 10.1016/j.brs.2018.10.017 PMC6347516

[109] Pittig A, Heinig I, Goerigk S, Richter J, Hollandt M, Lueken U, et al. Change of threat expectancy as mechanism of exposure-based psychotherapy for anxiety disorders: evidence from 8484 exposure exercises of 605 patients. Clin Psychol Sci. 2023;11:199–217.

[110] Effting M, Kindt M. Contextual control of human fear associations in a renewal paradigm. Behav Res Ther. 2007;45:2002–18. 17451643 10.1016/j.brat.2007.02.011

[111] Mineka S, Mystkowski JL, Hladek D, Rodriguez BI. The effects of changing contexts on return of fear following exposure therapy for spider fear. J Consult Clin Psychol. 1999;67:599–604. 10450633 10.1037//0022-006x.67.4.599

[112] Souza RR, Robertson NM, Pruitt DT, Gonzales PA, Hays SA, Rennaker RL, et al. Vagus nerve stimulation reverses the extinction impairments in a model of PTSD with prolonged and repeated trauma. Stress. 2019;22:509–20. 31010369 10.1080/10253890.2019.1602604

