# Supplementary material for: Ready for translation: non-invasive auricular vagus nerve stimulation inhibits psychophysiological indices of stimulus-specific fear and facilitates responding to repeated exposure in phobic individuals
Source: Transl Psychiatry. 2025 Apr 9;15:135. doi: 10.1038/s41398-025-03352-0 (PMC11982236; doi:10.1038/s41398-025-03352-0)
Supplement: Supplementary file 1 — Supplemental Material [file 41398_2025_3352_MOESM1_ESM.pdf]

Supplemental Results

Vagus nerve stimulation does not affect average heart rate during laboratory exposure in vitro.

To test, whether taVNS influenced mean heart rate, we performed a linear mixed model, that regressed mean heart rate (in bpm) during the four experimental *Blocks* (*Neutral, Exposed Tarantula, Spiders, Mixed*) onto the effect of *Stimulation*. There was no significant difference between the taVNS and sham condition with regard to mean heart rate across the four blocks (*Stimulation*, $F_{1,30}$ = 0.189, p = .667), indicating that vagal stimulation did not affect mean heart rate, but rather heart rate reactivity to fear-eliciting stimuli (see main manuscript).

Vagus nerve stimulation sensitizes startle magnitudes after participants were exposed to a living tarantula.

To test, whether taVNS affected startle magnitudes regardless of the presented visual stimuli, we performed a linear mixed model, that tested the between-group difference between both *Stimulation* conditions (taVNS vs. sham stimulation) for startle magnitudes elicited across all experimental *Blocks* (*Startle Habituation, Inter-trial Intervals, Neutral, Exposed Tarantula, Spiders, Mixed*). In comparison to the sham stimulation condition, startle reflex magnitudes were sensitized when participants received active vagus nerve stimulation during the laboratory exposure in vitro, immediately after they had been exposed to the real-life fear cue (tarantula) during the exposure in vivo I (*Stimulation,* $F_{1,30}$ = 7.183, p = .012; **Supplemental Figure S2**). Importantly, as this enhancement was also found for the startle responses elicited during baseline at the very beginning of the laboratory exposure, results suggest that startle reflex magnitudes were overall sensitized by vagal stimulation, with no additional activation of the participant’s defense system (e.g., by presenting pictures of spiders).

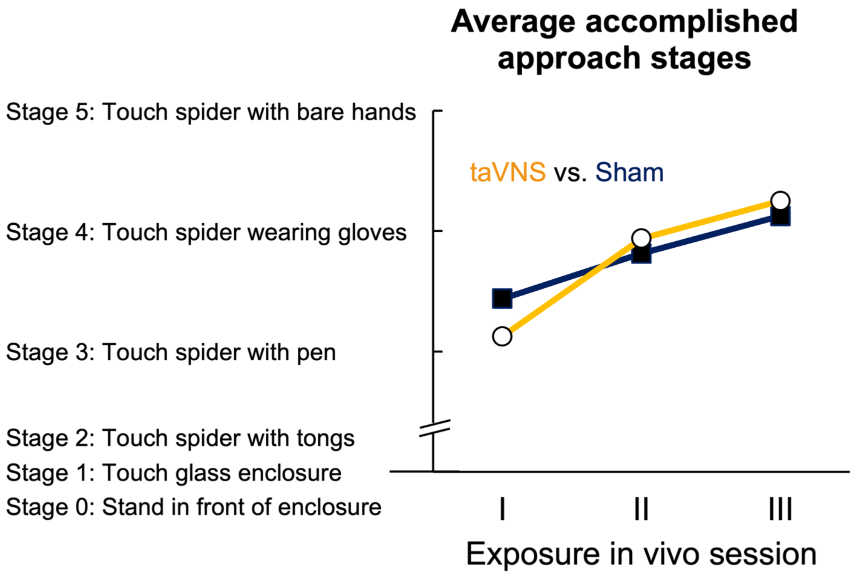

Supplemental Figure 1. Approach behavior is facilitated by repeated exposure in vivo sessions. Average approach stages accomplished on the behavioral approach task.

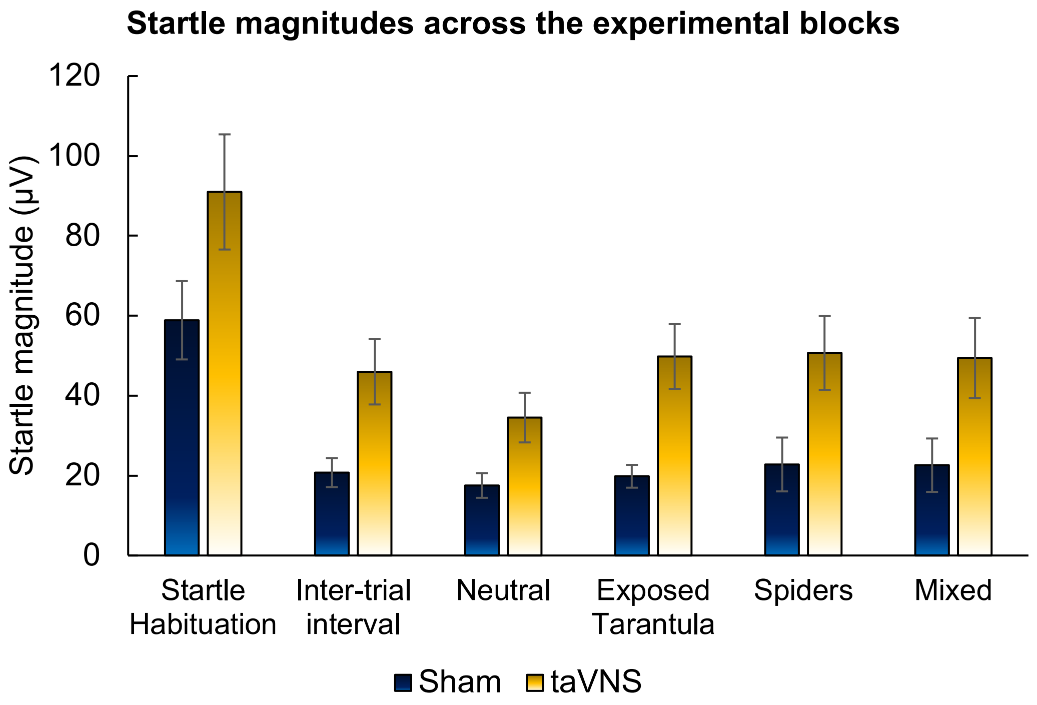

Supplemental Figure 2. Startle magnitudes are sensitized by vagal stimulation. Startle response magnitudes (µV) for each experimental block, including the startle habituation phase at the beginning of the experiment, for the sham (blue bars) and taVNS condition (orange bars). Error bars represent the standard error of the mean.

Overall

Fear index / Completer during in-vivo exposure	Exposure in vivo II	Exposure in vivo III
Mean heart rate increase during *Exposed Tarantula* block	*r* = -.097<br>*p* = .596	*r* = -.228<br>*p* = .209
Mean corrugator activity increase during *Exposed Tarantula* block	*r* = .097<br>*p* = .597	*r* = .091<br>*p* = .620
Mean overall startle response magnitude	*r* = .095<br>*p* = .603	*r* = .092<br>*p* = .616

Sham

Fear index / Completer during in-vivo exposure	Exposure in vivo II	Exposure in vivo III
Mean heart rate increase during *Exposed Tarantula* block	*r* = -.367<br>*p* = .162	*r* = -.481<br>*p* = .059
Mean corrugator activity increase during *Exposed Tarantula* block	*r* = -.007<br>*p* = .979	*r* = -.071<br>*p* = .793
Mean overall startle response magnitude	*r* = -.423<br>*p* = .102	*r* = -.667<br>*p* = .005

taVNS

Fear index / Completer during in-vivo exposure	Exposure in vivo II	Exposure in vivo III
Mean heart rate increase during *Exposed Tarantula* block	*r* = .264<br>*p* = .323	*r* = .116<br>*p* = .668
Mean corrugator activity increase during *Exposed Tarantula* block	*r* = .317<br>*p* = .231	*r* = .411<br>*p* = .114
Mean overall startle response magnitude	*r* = .368<br>*p* = .160	*r* = .745<br>*p* < .001

Supplemental Table 1. Fear responses during in-vivo and laboratory exposure towards the real tarantula are not related. Point-biserial correlations between mean heart rate/corrugator activity change during viewing of pictures of the previously exposed tarantula (*Exposed Tarantula* block in the laboratory exposure) and completers in the exposure in vivo II and III for the overall sample (upper table), the sham group (middle table) and taVNS group (lower table).
